# Origin Variants of the Ascending Pharyngeal Artery and Sequential External Carotid Branching Classification

**DOI:** 10.3390/diagnostics15243106

**Published:** 2025-12-06

**Authors:** Rodica Narcisa Calotă, Alexandra Diana Vrapciu, Sorin Hostiuc, Marius Ioan Rusu, Răzvan Costin Tudose, Mihail Silviu Tudosie, George Triantafyllou, Maria Piagkou, Mugurel Constantin Rusu

**Affiliations:** 1 Division of Anatomy, Department 1, Faculty of Dentistry, “Carol Davila” University of Medicine and Pharmacy, 050474 Bucharest, Romania; rodica-narcisa.plai@drd.umfcd.ro (R.N.C.); alexandra.vrapciu@umfcd.ro (A.D.V.); razvan-costin.tudose0721@stud.umfcd.ro (R.C.T.); mugurel.rusu@umfcd.ro (M.C.R.); 2 University Emergency Hospital Bucharest, 050098 Bucharest, Romania; 3 Division of Legal Medicine and Bioethics, Faculty of Dentistry, “Carol Davila” University of Medicine and Pharmacy, 050474 Bucharest, Romania; sorin.hostiuc@umfcd.ro; 4 Division of Economic Informatics, Faculty of Cybernetics, Statistics and Economic Informatics, University of Economic Studies, 010374 Bucharest, Romania; rusumarius21@stud.ase.ro; 5 Faculty of Medicine, “Carol Davila” University of Medicine and Pharmacy, 050474 Bucharest, Romania; 6 Department of Anatomy, School of Medicine, Faculty of Health Sciences, National and Kapodistrian University of Athens, 75 Mikras Asias Str., Goudi, 11527 Athens, Greece; georgerose406@gmail.com (G.T.); mapian@med.uoa.gr (M.P.)

**Keywords:** ascending pharyngeal artery, external carotid artery, anatomical variation, sequential classification, occipitopharyngeal trunk, carotid bifurcation, computed tomography angiography, bilateral assessment, head and neck surgery, vascular anatomy

## Abstract

**Background/Objectives:** The ascending pharyngeal artery (APA) exhibits considerable variability in origin. Understanding its anatomy is essential for head and neck surgery, endovascular procedures, and skull base approaches. This study aimed to (1) systematically characterize APA origin sites, (2) evaluate bilateral patterns, and (3) establish a comprehensive sequential classification system for external carotid artery (ECA) branching. **Methods:** Bilateral computed tomography angiography assessment was performed in 85 patients (170 carotid axes; 54 men, 31 women; mean age 69 ± 10 years). APA origins were classified into six types: Type 0 (absent), Type I (ECA medial wall), Type II (ECA posterior wall), Type III (occipitopharyngeal trunk), Type IV (internal carotid artery), and Type V (other origins). A novel sequential classification system (S-types) documented the complete ECA branching order. **Results:** APA was absent in 14.71% of cases; APA’s absence or internal carotid origin was noted in 19.41% of cases. Type I occurred in 26.47%, Type II in 35.88%, Type III in 17.06%, Type IV in 4.71%, and Type V in 1.18%. Forty distinct S-types were identified, representing the most comprehensive documentation of ECA branching diversity. No statistically significant side-related (χ^2^ = 42.12, *p* = 0.379) or gender-related (χ^2^ = 49.81, *p* = 0.138) differences were found. Twenty-three types occurred in fewer than five cases each. **Conclusions:** This first comprehensive sequential classification system reveals extraordinary anatomical diversity in ECA branching patterns. The absence of predictable side or gender patterns necessitates bilateral preoperative imaging for surgical planning.

## 1. Introduction

In the neck, the common carotid artery (CCA) bifurcates (CB, carotid bifurcation) into the internal (ICA) and external (ECA) carotid arteries. Further, the ECA sends off six collateral branches. They are the superior thyroid (STA), lingual (LA), facial (FA), ascending pharyngeal (APA), occipital (OA), and posterior auricular (PAA) arteries. The APA is a small but anatomically significant branch of the ECA. It may originate in a common trunk with the OA—the occipitopharyngeal trunk (OPT). Additionally, other branches of the ECA may branch off into common trunks, including the thyrolingual trunk (TLT), linguofacial trunk (LFT), or thyrolinguofacial trunk.

In the head and neck, the APA is one of the arteries without a cutaneous territory [1]. As noted in Bergman’s Encyclopedia of Human Anatomic Variations, the APA leaves the ECA as the first or second branch and ascends deep to the ICA, passing medially to the tensor veli palatini muscle [2,3]. Thus, the APA courses into the parapharyngeal space [2]. The APA vascularizes the last four cranial nerves, anastomoses with the vertebrobasilar system, and may vascularize various tumors of the parapharyngeal space [2]. It may serve as a landmark to identify the parapharyngeal segment of the ICA [2].

As noted in Bergman’s Encyclopedia of Human Anatomic Variations, the APA typically leaves the ECA as the first or second branch [2,3]. It ascends deep to the ICA, passing medially to the tensor veli palatini muscle, and courses into the parapharyngeal space [2]. The APA vascularizes the last four cranial nerves and anastomoses with the vertebrobasilar system [2]. It may also supply the various parapharyngeal space tumors and serves as a landmark to identify the parapharyngeal segment of the ICA [2].

Variants of the APA are uncommon. Variant origins from the ICA or CCA are rare (0.14–5%). The APA originates from the ECA in about 80% of cases. The origin of APA from the ICA was reported in 0.14% to 5% of cases, depending on the study and population [4,5,6,7]. ICA-origin APA shows variable prevalence: one cadaveric study reported 5% [5,6], while an extensive dissection study found only 0.14% [4]. Other variant origins include the CCA bifurcation, OA, and linguofacial trunk (LFT). These variants each occurred in approximately 5% of cadaveric specimens [5,6]. APA origin from the FA occurs in 2% of cases [8]. Other rare variants include APA supplying the posterior inferior cerebellar artery via unusual anastomoses or branching patterns involving the hypoglossal canal or jugular foramen. Still, these are extremely rare and mostly described in isolated case reports [7,9,10,11,12].

Understanding APA anatomical variations is crucial for multiple clinical scenarios. These include procedures involving the carotid space, skull base surgery, and assessment of vascular supply to the pharynx and meninges. Despite its small caliber, the APA’s extensive distribution makes it clinically relevant in various pathological conditions and surgical approaches. The anatomy of the APA can show considerable individual variation, particularly in branching patterns and the specific territories supplied by each branch. Therefore, recognition of APA variants is crucial for head and neck surgery, endovascular procedures, and radiological planning to avoid complications [4,5,6,7,13,14]. Imaging (CT/MR angiography) is recommended preoperatively to identify these variants [4,7,10,13].

The extraordinary variability in ECA branching patterns has embryological foundations in the complex remodeling of the aortic arch system during the 4th to 8th gestational weeks. The ECA and its branches derive from the ventral pharyngeal arteries and third aortic arch derivatives, with the APA specifically originating from the cranial extension of the dorsal aortic root [15]. During embryogenesis, the paired aortic arches undergo asymmetric regression, persistence, and fusion patterns, with selective segments retained or obliterated in highly variable configurations [16]. The sequential branching order of ECA collaterals reflects the timing and spatial positioning of pharyngeal arch artery sprouts, which emerge from the ventral aorta in a craniocaudal sequence but may persist, regress, or anastomose with neighboring vessels in individualized patterns [17]. Variations in the regression timing of embryonic vascular plexuses, combined with persistence of normally transient anastomoses between pharyngeal arch derivatives, account for the diverse origin sites and common trunk formations observed in adult anatomy [18]. This developmental stochasticity, occurring without systematic lateralization or sexual dimorphism, creates the anatomical substrate for the branching diversity documented in adult populations.

The recognized variability in APA origin suggests corresponding variation in ECA sequential branching patterns. We hypothesized that the complete branching sequence of the ECA’s initial branches would demonstrate extraordinary anatomical diversity. This study, therefore, aimed to (1) systematically characterize APA origin sites, (2) evaluate bilateral symmetry and asymmetry patterns, and (3) establish a sequential classification system for ECA proximal branches relative to APA origin.

## 2. Materials and Methods

Determinations were performed in a group of 85 archived angio-CT cases comprising 54 men and 31 women, with a mean age of 69 ± 10 years. The scans were conducted between July 2024 and January 2025. The study adhered to the principles outlined in the Declaration of Helsinki. The responsible authorities (affiliation 2) approved the study (approval no. 10540/16 February 2022).

Good-quality, contrast-enhanced scans of the carotid arteries and their branches, covering both the neck and the head, were included in the study, provided that they did not contain pathologic processes that distorted the arterial anatomy. Exclusion criteria were poor scan quality, incomplete contrast injection of the arteries, pathologic processes, and previous neck surgery. The scans were initially performed for clinical indications unrelated to parapharyngeal, vascular, or cervical pathology, but to follow or evaluate the cerebral circulation. No cases were excluded.

Angio-CT imaging was performed using a 32-slice Siemens Multislice Perspective Scanner (Forcheim, Germany) with 0.6 mm collimation, 0.75 mm slice thickness, and 50% overlap. Maximum-intensity projection and three-dimensional volume rendering techniques were applied according to established protocols [19]. Image analysis and documentation were performed using Horos software version 3.3.6 for macOS (Horos Project, Annapolis, MD, USA), as utilized in previous studies [20]. Anatomical assessments were confirmed through two-dimensional planar reconstructions and recorded using three-dimensional volume-rendered images. Each author conducted independent evaluations of the carotid arterial system. Positive findings demonstrated complete concordance among all reviewers and underwent validation by each investigator.

We recorded the uni- and bilateral absence of APA. We classified the origin of the APA into the following types: type I—origin from the medial wall of the ECA (ECA-M); type II—origin from the posterior wall of the ECA (ECA-P); type III—origin from the OPT; type IV—origin from the ICA; and type V—other origins. The absence of APA was recorded as type 0. We referred the origin of the APA to the greater hyoid horn and classified it into suprahyoid (SH), hyoid (H), and infrahyoid (IH). We also recorded the origin of the APA and OPT in the sequence of the first branches of the ECA and defined the ‘S’ types; in the situation of absence of APA or its origin from the ICA, we recorded it as type S0. Note that S0 includes both absent APA (Type 0) and ICA-origin APA (Type IV), as both represent cases where APA did not arise from the ECA in the standard branching sequence.

Microsoft Excel was utilized for preliminary data analysis to obtain an initial overview of the collected data. Subsequent statistical analyses, including multiple and straightforward regression tests and analysis of variance (ANOVA), were conducted using EViews 12 statistical software. Continuous variables were analyzed across groups (gender, laterality, APA origins and trajectories, hyoid bone ratios). Regression analysis was employed to determine associations between these variables and their mutual influences. Statistical significance was defined as *p* < 0.05.

## 3. Results

### 3.1. The Origin of the APA

**Figure 1:**
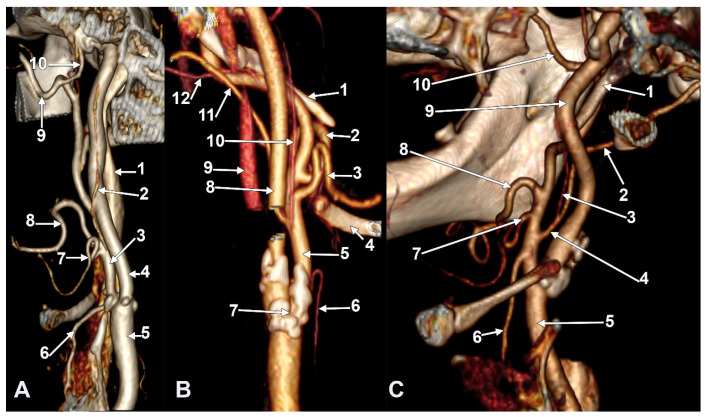
(**A**) Type I ascending pharyngeal artery originating from the medial wall of the external carotid artery. Three-dimensional rendering. Right side. Anteromedial view. 1. Internal jugular vein; 2. ascending pharyngeal artery; 3. external carotid artery; 4. internal carotid artery; 5. common carotid artery; 6. superior thyroid artery; 7. lingual artery; 8. facial artery; 9. maxillary artery; 10. middle meningeal artery. (**B**) Type II ascending pharyngeal artery originating from the posterior wall of the external carotid artery. Three-dimensional rendering. Left side. Posteromedial view. 1. Styloid process; 2. facial artery; 3. lingual artery; 4. greater hyoid horn; 5. external carotid artery; 6. superior thyroid artery; 7. carotid bifurcation; 8. internal carotid artery; 9. internal jugular vein; 10. ascending pharyngeal artery; 11. occipital artery; 12. posterior auricular artery. (**C**) Type III ascending pharyngeal artery originating from the occipitopharyngeal trunk. Three-dimensional rendering. Right side. Anteromedial view. 1. Styloid process; 2. occipital artery; 3. ascending pharyngeal artery; 4. occipitopharyngeal trunk; 5. common carotid artery; 6. superior thyroid artery; 7. lingual artery; 8. facial artery; 9. internal carotid artery; 10. maxillary artery.

**Figure 2:**
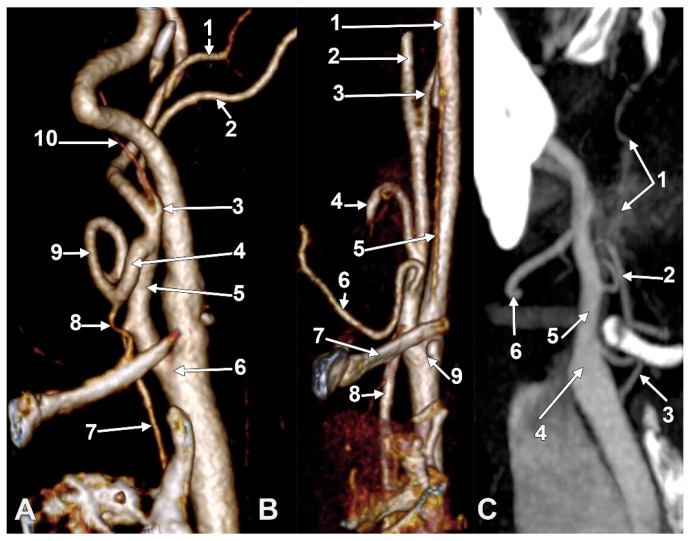
(**A**) Type III ascending pharyngeal artery originating from the occipitopharyngeal trunk. Linguofacial trunk. Three-dimensional rendering. Right side. Anteromedial view. 1. Posterior auricular artery; 2. occipital artery; 3. occipitopharyngeal trunk; 4. linguofacial trunk; 5. external carotid artery; 6. carotid bifurcation; 7. superior thyroid artery; 8. lingual artery; 9. facial artery; 10. ascending pharyngeal artery. (**B**) Type IV ascending pharyngeal artery originating from the internal carotid artery. Three-dimensional rendering. Right side. Anteromedial view. 1. Internal carotid artery; 2. external carotid artery; 3. occipital artery; 4. facial artery; 5. ascending pharyngeal artery; 6. lingual artery; 7. greater hyoid horn; 8. superior thyroid artery; 9. carotid bifurcation. (**C**) Type V ascending pharyngeal artery originating from the lingual artery. Right side. Coronal slice. Anterior view. 1. Ascending pharyngeal artery; 2. lingual artery; 3. superior thyroid artery; 4. carotid bifurcation; 5. external carotid artery; 6. facial artery.

We investigated 170 carotid axes bilaterally. The APA exhibited six origin patterns: Type 0 (absent) in 25 sides (14.71%), Type I (ECA medial wall, Figure 1A) in 45 sides (26.47%), Type II (ECA posterior wall, Figure 1B) in 61 sides (35.88%), Type III (occipitopharyngeal trunk, Figure 1C and Figure 2A) in 29 sides (17.06%), Type IV (ICA origin, Figure 2B) in 8 sides (4.71%), and Type V (other origins including LA—Figure 2C—and CB) in 2 sides (1.18%). For sequential classification purposes, Types 0 and IV were designated as S0 (33 sides, 19.41%) because both represent the absence of APA from the ECA branching sequence.

We detailed the origin types of APA on the right and left sides (Figure 1 and Figure 2, Table 1), respectively, in the investigated cohort (*N* = 85). On the right side, we found types 0, I, II, III, IV, and V, with prevalences of 12.9%, 37.6%, 24.7%, 14.1%, 9.4%, and 1.2%, respectively. On the left, the prevalence of types 0–V was in the following order: 16.5%, 15.3%, 47.1%, 20.0%, 0.0%, and 1.2%.

**Table 1:** Summary of anatomical variants illustrated by figure panels. Abbreviations: APA = ascending pharyngeal artery; ECA = external carotid artery; ICA = internal carotid artery; OPT = occipitopharyngeal trunk; LFT = linguofacial trunk; TLT = thyrolingual trunk; CB = carotid bifurcation; IPS = inferior petrosal sinus; STA = superior thyroid artery; IH = infrahyoid; H = hyoid level; SH = suprahyoid.

Figure	Panel(s)	Variant(s) Illustrated	Prevalence	Rarity
Figure 1A	A	Type I APA (ECA medial wall)	26.47%	Common
Figure 1B	B	Type II APA (ECA posterior wall)	35.88%	**Most common**
Figure 1C	C	Type III APA (from OPT)	17.06%	Common
Figure 2A	A	Type III APA + LFT	17.06%	Common
Figure 2B	B	Type IV APA (from ICA)	4.71%	Rare
Figure 2C	C	Type V APA (from LA)	1.18%	Very rare
Figure 3A	A	Infrahyoid (IH) APA + LFT	8.24%	Uncommon
Figure 3B	B	Hyoid-level (H) APA	5.29%	Uncommon
Figure 3C	C	S37 type + OPT + absent STA	1.18%	Very rare
Figure 4	-	Reference diagram (no variants)	N/A	N/A
Figure 5	-	S-types 1–20 (schematic)	Mixed	S7: 7.1%
Figure 6	-	S-types 21–40 (schematic)	Mixed	Multiple rare
Figure 7	-	Long IPS + Type IV APA + long styloid	Case report	Very rare
Figure 8	A, B	Same case as Figure 7 (3D views)	Case report	Very rare
Figure 9	A, B	TLT from CCA (S33 pattern)	1.18%	Very rare
Figure 10	A, B	TLT from ECA (S35) + collapsed hyoid	1.18%	Very rare
Figure 11	A, B	Type V from CB + S40 + multiple variants	1.18%	Very rare

**Note:** Figure 1, Figure 2 and Figure 3 illustrate the six APA origin types (Types 0, I, II, III, IV, V) and hyoid topography variants. Figure 5 and Figure 6 show all 40 S-type sequential patterns schematically. Figure 7, Figure 8, Figure 9, Figure 10 and Figure 11 present detailed case examples of rare variants, including TLT, long IPS, and complex S-type patterns. S37 = STA absent-OPT-LA-FA; S33 = TLT (from CCA)-APA-FA-OA; S35 = TLT (from ECA)-OA-APA-FA; S40 = STA/APA from CB-OA/SCM br.-LA-FA.

**Figure 3:**
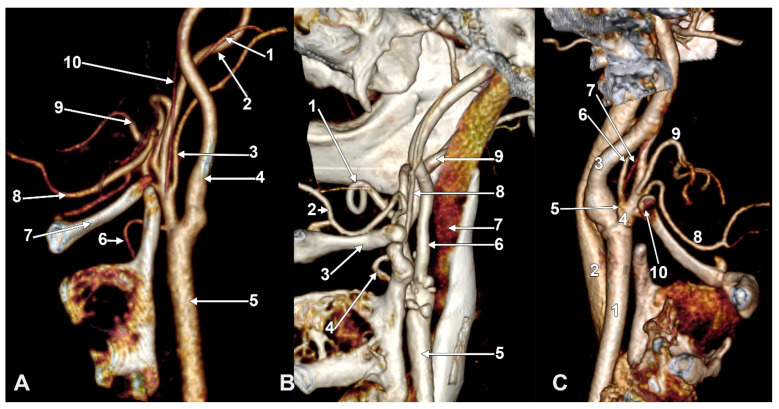
(**A**) Infrahyoid origin of the ascending pharyngeal artery. Linguofacial trunk. Three-dimensional rendering. Antero-infero-medial view. 1. Posterior auricular artery; 2. external carotid artery; 3. occipital artery; 4. internal carotid artery; 5. common carotid artery; 6. superior thyroid artery; 7. greater hyoid horn; 8. lingual artery; 9. facial artery; 10. ascending pharyngeal artery. (**B**) The hyoid origin of the ascending pharyngeal artery, lateral to the greater hyoid horn. Three-dimensional volume rendering. Right side. Posteromedial view. 1. Facial artery; 2. lingual artery; 3. greater hyoid horn; 4. superior thyroid artery; 5. common carotid artery; 6. internal carotid artery; 7. internal jugular vein; 8. ascending pharyngeal artery; 9. external carotid artery. (**C**) Occipitopharyngeal trunk and absent superior thyroid artery. Type S 37 of sequence of the origin of the external carotid branches. Three-dimensional rendering. Left side. Medial view. 1. Common carotid artery; 2. internal jugular vein; 3. internal carotid artery; 4. external carotid artery; 5. occipitopharyngeal trunk; 6. occipital artery; 7. ascending pharyngeal artery; 8. lingual artery; 9. facial artery; 10. hyoid tubercle.

**Figure 4:**
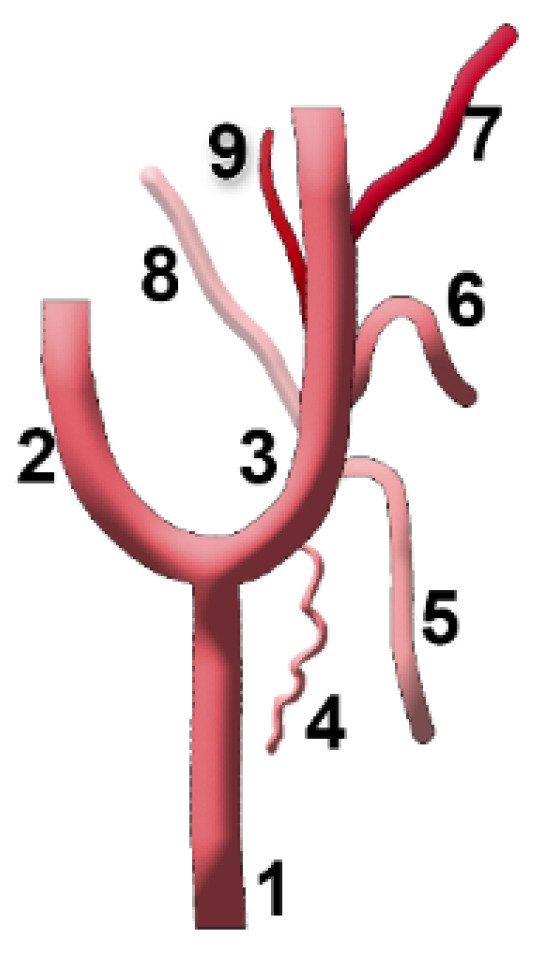
Anatomical diagram used to represent the types of sequence of the origin of the ascending pharyngeal artery from the external carotid artery. 1. Common carotid artery (CCA); 2. internal carotid artery (ICA); 3. external carotid artery (ECA); 4. sternocleidomastoid (SCM) br.; 5. superior thyroid artery (STA); 6. lingual artery (LA); 7. facial artery (FA); 8. occipital artery (OA); 9. ascending pharyngeal artery (APA).

**Figure 5:**
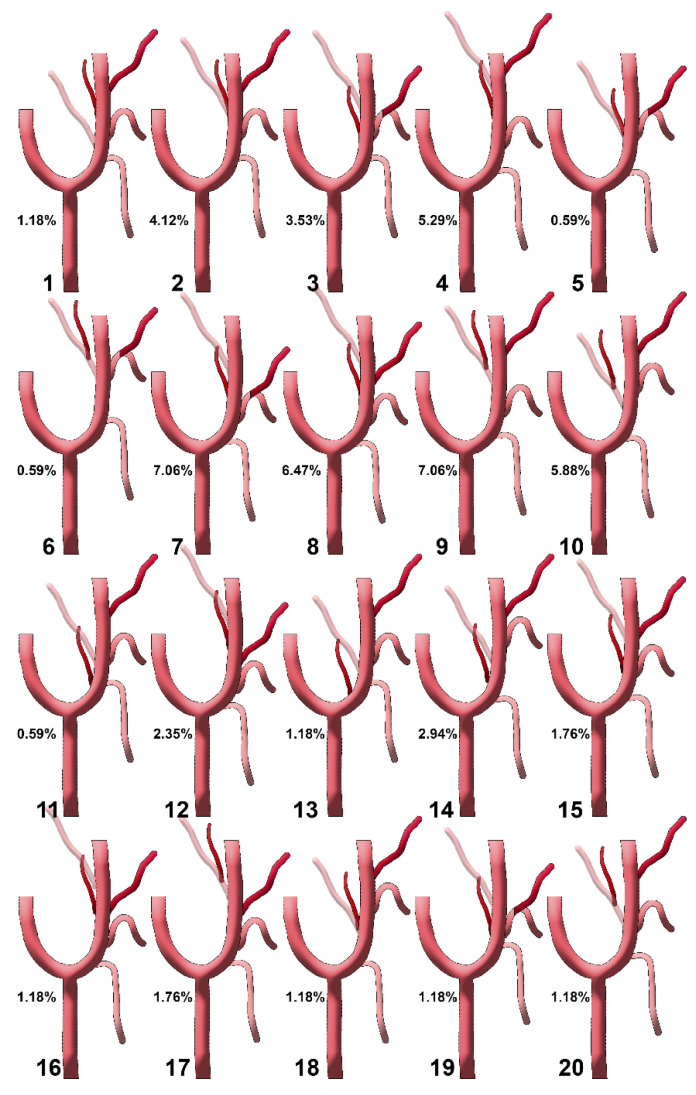
Diagrams of sequence types 1–20 of the origin of the ascending pharyngeal artery from the external carotid artery. Their prevalences are shown. Type 1: STA-OA-LA-APA-FA; type 2: STA-LA-APA/OA-FA; type 3: STA-APA/LFT-OA; type 4: STA-LA-APA-OA-FA; type 5: STA-APA/LFT/OA-FA; type 6: STA-LFT/OPT; type 7: STA-LFT-APA-OA; type 8: STA-LA-APA-FA-OA; type 9: STA-LA-OPT-FA; type 10: STA-OPT-LA-FA; type 11: STA/APA-LA/OA-FA; type 12: STA-LA-FA-APA-OA; type 13: APA-STA-OA-LA-FA; type 14: STA-APA-OA-LA-FA; type 15: STA-LA/APA-OA/FA; type 16: STA-LA-APA/FA-OA; type 17: STA-LA-FA-OPT; type 18: STA-APA/OA-LA-FA; type 19: STA-LFT/APA-OA; type 20: STA-OPT/LA-FA. STA: superior thyroid artery; LA: lingual artery; FA: facial artery; OA: occipital artery; APA: ascending pharyngeal artery; OPT: occipitopharyngeal trunk; LFT: linguofacial trunk.

**Figure 6:**
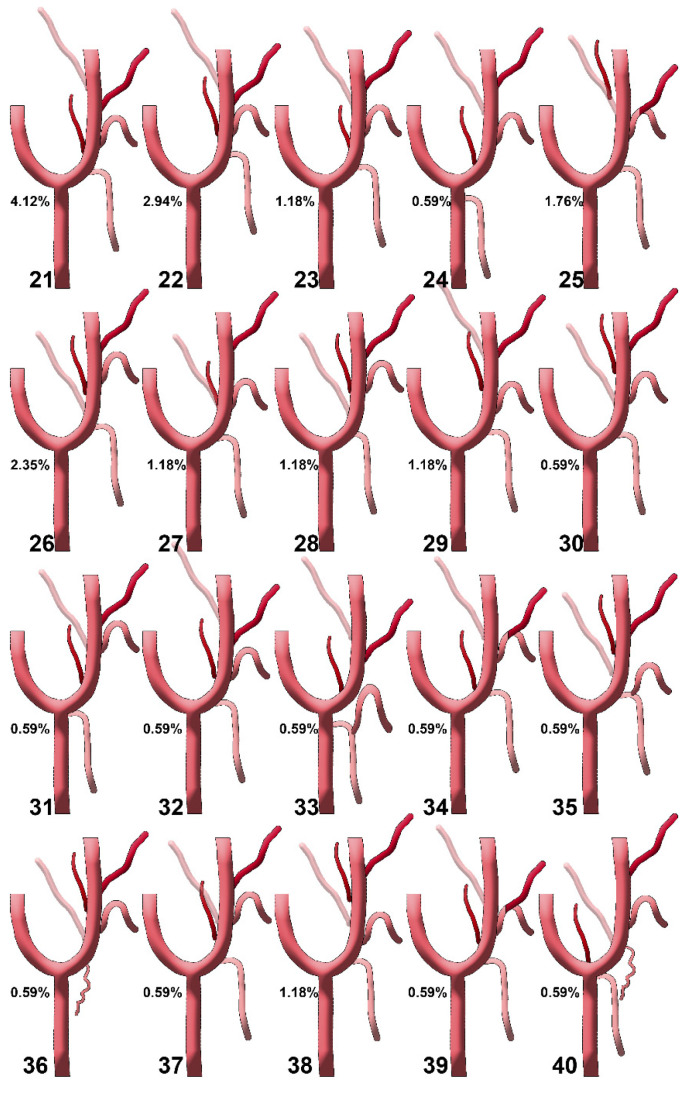
Diagrams of sequence types 21–40 of the origin of the ascending pharyngeal artery from the external carotid artery. Their prevalences are shown. Type 21: STA-APA-LA-FA-OA; type 22: STA-LA/APA-FA-OA; type 23: STA-APA-LA-OA-FA; type 24: APA-LA-OA-FA (STA from CCA); type 25: STA-LFT-OPT; type 26: STA-OA-APA-LA-FA; type 27: STA-LA/APA/OA-FA; type 28: STA-OA-LA/APA-FA; type 29: STA-LA-APA-FA/OA; type 30: STA-LA/OA-APA-FA; type 31: STA (from CB)-APA-LA/OA-FA; type 32: STA-APA/LA-FA-OA; type 33: TLT (from CCA)-APA-FA-OA; type 34: STA/APA-LFT-OA; type 35: TLT (from ECA)-OA-APA-FA; type 36: (S0, STA absent or with internal carotid origin)-SCM br.-OA-LA-APA-FA; type 37: STA-APA-LA/OA-FA; type 38: STA-LA-OA-APA-FA; type 39: STA-APA-LFT-OA; type 40: STA/APA from CB-OA/SCM br.-LA-FA. STA: superior thyroid artery; LA: lingual artery; FA: facial artery; OA: occipital artery; APA: ascending pharyngeal artery; CCA: common carotid artery; CB: carotid bifurcation; CCA: common carotid artery; ECA: external carotid artery; OPT: occipitopharyngeal trunk; LFT: linguofacial trunk; TLT: thyrolingual trunk; SCM br.: sternocleidomastoid branch.

**Figure 7:**
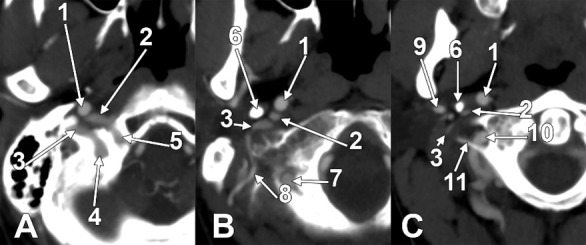
Successive axial sections from superior to inferior (**A**–**C**) through the long inferior petrosal sinus. Right side. Inferior views. 1. Internal carotid artery; 2. inferior petrosal sinus; 3. internal jugular vein; 4. sigmoid sinus; 5. anterior condylar vein; 6. styloid process; 7. vertebral artery; 8. occipital artery; 9. external carotid artery; 10. vertebral artery; 11. transverse process of the atlas.

**Figure 8:**
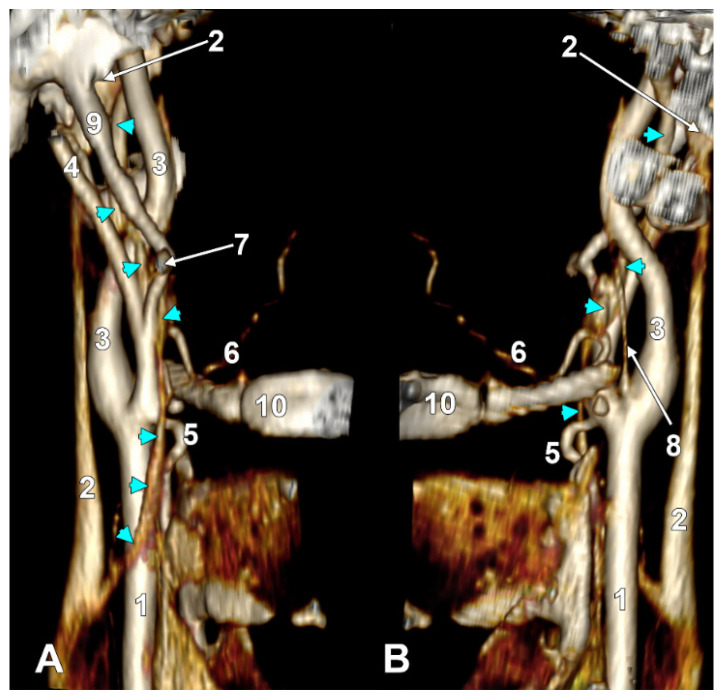
Long inferior petrosal sinus (arrowheads). Internal carotid origin of the ascending pharyngeal artery. Right side. (**A**) Anterolateral view. (**B**) Anteromedial view. 1. Common carotid artery; 2. internal jugular vein; 3. internal carotid artery; 4. external carotid artery; 5. superior thyroid artery; 6. lingual artery; 7. facial artery; 8. ascending pharyngeal artery; 9. styloid process; 10. body of the hyoid.

**Figure 9:**
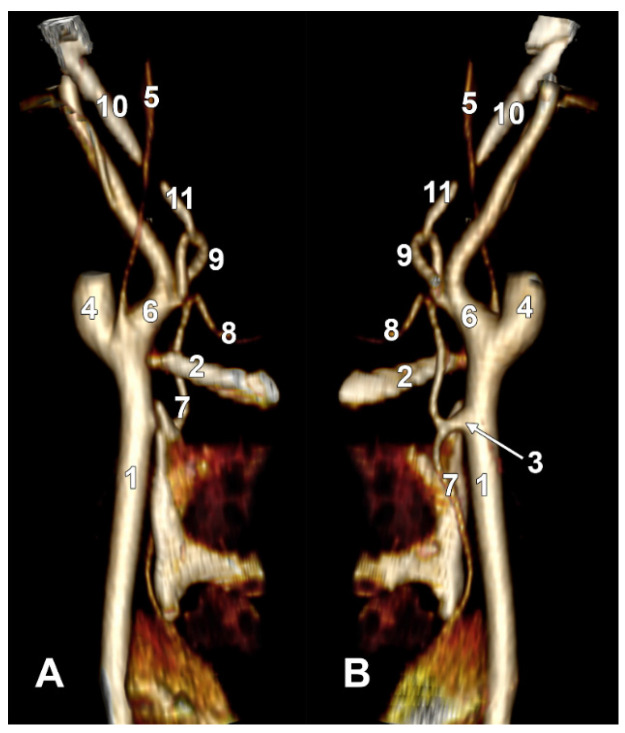
Thyrolingual trunk originating from the common carotid artery. The ascending pharyngeal artery is the first branch of the external carotid artery. Type 33 of the sequence of branches of the external carotid artery. Three-dimensional rendering. Left side. (**A**) Posteromedial view. (**B**) Antero-lateral view. 1. Common carotid artery; 2. greater hyoid horn; 3. thyrolingual trunk; 4. internal carotid artery; 5. ascending pharyngeal artery; 6. external carotid artery; 7. superior thyroid artery; 8. lingual artery; 9. facial artery; 10. styloid process; 11. ossified stylohyoid ligament.

**Figure 10:**
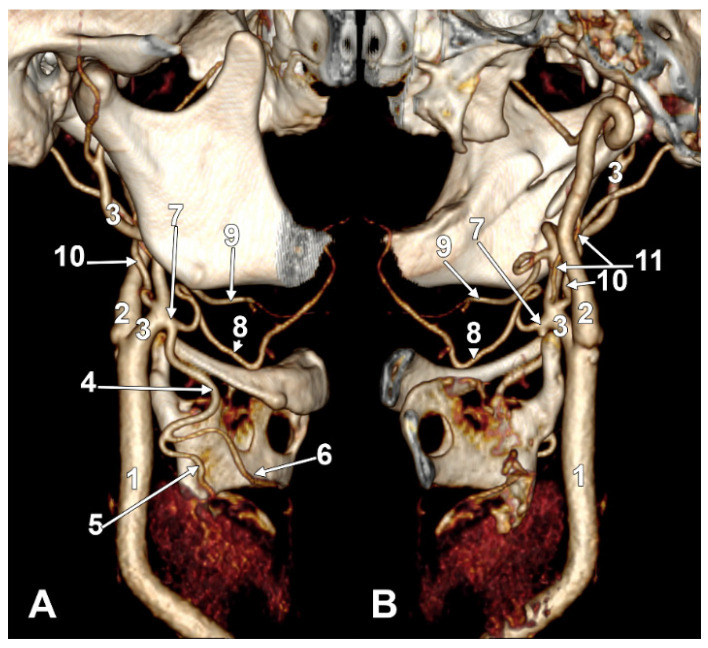
S-type 35 of the sequence of the external carotid artery branches. The hyoid collapsed over the thyroid cartilage of the larynx, as reported in [21]. Right side. (**A**) Lateral view. (**B**) Medial view. 1. Common carotid artery; 2. internal carotid artery; 3. external carotid artery; 4. superior thyroid artery; 5. right thyroid branch; 6. left thyroid branch; 7. thyrolingual trunk; 8. lingual artery; 9. facial artery; 10. occipital artery; 11. ascending pharyngeal artery.

**Figure 11:**
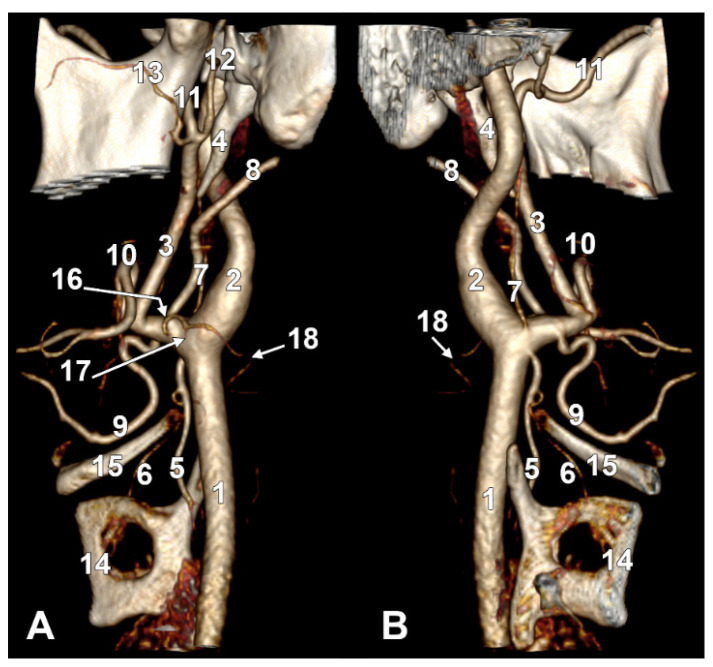
Type V ascending pharyngeal artery originating from the carotid bifurcation. Superior laryngeal artery with suprahyoid origin. Three-dimensional rendering. Left side. (**A**) Lateral view. (**B**) Medial view. 1. Common carotid artery; 2. internal carotid artery; 3. external carotid artery; 4. styloid process; 5. superior thyroid artery; 6. superior laryngeal artery; 7. ascending pharyngeal artery; 8. occipital artery; 9. lingual artery; 10. facial artery; 11. maxillary artery; 12. superficial temporal artery; 13. transverse facial artery; 14. thyroid cartilage; 15. greater hyoid horn; 16. sternocleidomastoid branch; 17. descending branch; 18. posterior branch.

The distribution of APA origin types was analyzed by gender. Bilaterally in men (*N* = 108), there were 16 (14.8%) absent APAs, 26 (24.1%) type I APAs, 43 (39.8%) type II APAs, 17 (15.7%) type III APAs, 5 (4.6%) type IV APAs, and 1 (0.9%) type V APA. Bilaterally in women (*N* = 62), there were 9 (14.5%) absent APAs, 19 (30.6%) APA type I, 18 (29%) APA type II, 12 (19.4%) APA type III, 3 (4.8%) APA type IV and 1 (1.6%) APA type V. The distribution of types 0–V on each side of the median plane by gender is shown in Table 2.

### 3.2. Bilateral Combinations of the Origin Types of the APA

The following bilateral combinations of APA origin types resulted: 0-0 (abs/abs), I-0 (ECA-M/abs), I-II (ECA-M/ECA-P), I-I (ECA-M/ECA-M), I-III (ECA-M/OPT), II-0 (ECA-P/abs), II-I (ECA-P/ECA-M), II-II (ECA-P/ECA-P), II-V (ECA-P/CB), II-III (ECA-P/OPT), IV-I (ACI/ECA-M), IV-II (ACI/ECA-P), IV-III (ACI/OPT), V-II (LA/ECA-P), III-0 (OPT/abs), III-II (OPT/ECA-P), III-III (OPT/OPT). Their count and prevalence are presented in Table 3.

### 3.3. The Hyoid Level of the APA’s Origin (Types SH/H/IH)

In the overall group of 170 sides, the APA originated inferior to the hyoid (IH type) in 8.24% of cases, at the hyoid level (H type) in 5.29% of cases, and suprahyoid (SH type) in 71.76% of cases, and was absent in the remaining sides. On the right side, the APA origin was infrahyoid in 8.24% (Figure 3A), hyoid in 7.06% (Figure 3B), and suprahyoid in 71.76% of cases, the rest being absent. On the left side, the APA origin was infrahyoid in 8.24%, hyoid in only 3.53% and suprahyoid in 71.76% of cases, the rest being absent.

### 3.4. The Occipitopharyngeal Trunk

In 29/170 (17.06%) of sides, we found a type III origin of APA, namely OPT. In the investigated group (*N* = 85), an OPT was identified in 14.12% (12/85) of cases on the right side. On the left side, we found an OPT in 20% (17/85).

#### 3.4.1. The Hyoid Types of the Occipitopharyngeal Trunk

The origin of OPT, relative to the hyoid, was bilateral (*N* = 170): hyoid (H) in 2/170 (1.18%), infrahyoid (IH) in 2/170 (1.18%), and suprahyoid (SH) in 25/170 (14.71%). On the right side, the values recorded for the vertical level of OPT origin were as follows: hyoid level (H)—1/85 (1.18%), infrahyoid level (IH)—1/85 (1.18%), and suprahyoid (SH)—10/85 (11.76%). For the left side, the respective values were 1/85 (1.18%)—H, 1/85 (1.18%)—IH, and 15/85 (17.65%)—SH.

#### 3.4.2. Origin of the Occipitopharyngeal Trunk

In all cases in which we identified the presence of an OPT, it originated from the ECA.

#### 3.4.3. The “S” Types with Occipitopharyngeal Trunk

In the overall batch of laterals (*N* = 170), OPT was identified in the following carotid branch sequence “S” types: types 6 (ATyS-TLF/OPT), 9 (ATyS-AL-OPT), 10 (ATyS-OPT-AL), 17 (ATyS-AL-AF-OPT), 20 (ATyS-OPT/AL-AF), 25 (ATyS-TLF-OPT), and 37 (ATyS absent, AL/OPT-AF) (Table 3, Figure 3C). On the right side, we found sequences with OPT as follows: 5 cases with type 10, 1 case with type 25, and 6 cases with type 9 (Table 3). On the left side, we found sequences with OPT as follows: 4 cases with type 10, 3 cases with type 17, 1 case with type 20, 2 cases with type 25, 1 case with type 37, 1 case with type 6, and 5 cases with type 9 (Table 4).

The distribution of type S branching patterns from the ECAs OPTs varied by gender and side. Type 9 was the most frequently observed pattern, occurring in 5 males and 1 female on the right side, and in 4 males and 1 female on the left side, representing a total prevalence of 12.9% (11/85 sides). Type 10 was present in 2 males and 3 females on the right side, and in 3 males and 1 female on the left side, with a prevalence of 10.6% (9/85 sides). Type 17 was exclusively found on the left side in three males, resulting in a prevalence of 3.5% (3/85 sides). Type 20 appeared only in 1 female on the left side (1.2% prevalence). Type 25 was documented in 1 female on the right side and 2 females on the left side, with a prevalence of 3.5% (3/85 sides). Type 37 was identified in 1 female on the left side only (1.2% prevalence). Type 6 was observed in 1 female on the left side (1.2% prevalence). Overall, types 9 (12.9%) and 10 (10.6%) demonstrated the highest prevalence, accounting for 23.5% of all cases, while the remaining patterns (types 17, 20, 25, 37, and 6) were rare, collectively representing 10.6% of cases, and showed sex-specific and side-specific distributions.

### 3.5. Framing of the Ascending Pharyngeal Artery in the Sequence of Origin of the First Branches of the External Carotid Artery—The S-Types

We recorded the sequence of origin from the ECA of the initial consecutive branches (as diagrammed in Figure 4), and found 40 different anatomical sequences (‘S’-types, Figure 5 and Figure 6): type 1—STA-OA-LA-APA-APA-FA, type 2—STA-LA-APA-APA/OA-FA (*/* refers to the same-level origin of the respective arteries), type 3—STA-APA/LFT-OA, type 4—STA-LA-APA-APA-OA-FA, type 5—STA-APA/LFT/OA-FA, type 6—STA-LFT/OPT, type 7—STA-LFT-APA-OA, type 8—STA-LA-APA-APA-FA-OA, type 9—STA-LA-OPT-FA, type 10—STA-OPT-LA-FA, type 11—STA/APA-LA/OA-FA, type 12—STA-LA-FA-APA-OA, type 13—APA-STA-OA-LA-FA type 14—STA-APA-APA-OA-LA-FA, type 15—STA-LA/APA-OA/FA, type 16—STA-LA-APA/FA-OA, type 17—STA-LA-FA-OPT, type 18—STA-APA-APA/OA-LA-FA, type 19—STA-LFT/APA-OA-OA, type 20—STA-OPT/LA-FA, type 21—STA-APA-LA-FA-OA, type 22—STA-LA/APA-FA-OA, type 23—STA-APA-LA-OA-FA, type 24—APA-LA-OA-FA (STA from ACC), type 25—STA-LFT-OPT, type 26—STA-OA-APA-LA-FA, type 27—STA-LA/APA/OA-FA, type 28—STA-OA-LA/APA-APA-FA, type 29—STA-LA-APA-APA-FA/OA, type 30—STA-LA/OA-APA-FA, type 31—STA (from BC)-APA-LA/OA-FA, type 32—STA-APA/LA-FA-OA, type 33—TLT (from CCA), APA-FA-OA, type 34—STA/APA-LFT-OA, type 35—TLT (from ECA)-OA-APA-APA-FA, type 36—(absent STA) sternocleidomastoid (SCM) br.-OA-LA-APA-FA, type 37—STA-APA-LA/OA-FA, type 38—STA-LA-OA-APA-FA, type 39—STA-APA-APA-LFT-OA, type 40—STA/APA from BC, OA/r.SCM-LA-FA.

On the right side, we did not identify S-types 5, 13, 16, 18, 23, 26, 30, 34, and 35. Types 6, 11, 15, 17, 20, 24, 29, 31, 32, 33, 36, 37, 39, and 40 were missing on the left side. In the overall batch of 170 sides, we found ‘S’ types 1 to 40 with different prevalences (Table 4). We determined the ‘S’ types of the sequence of the origin of the external carotid branches on either side of the median plane, right and left (Table 5).

The most common S-types (*N* = 170) were type 7: 12 cases (7.1%)—STA-LFT-APA-OA; type 9: 12 cases (7.1%)—STA-LA-OPT-FA; type 8: 11 cases (6.5%)—STA-LA-APA-FA-OA; type 10: 10 cases (5.9%)—STA-OPT-LA-FA; and type 4: 9 cases (5.3%)—STA-LA-APA-OA-FA.

Some S-types were side-specific: (A) right-only: types 5, 13, 16, 18, 23, 26, 30, 34, 35, and (B) left-only: types 6, 11, 15, 17, 20, 24, 29, 31, 32, 33, 36, 37, 39, 40. Rare variants documented include TLT from CCA (type 33) and TLT from ECA (type 35).

The gender distribution of ‘S’ types on the right side is shown in Table 5. In males, types 5, 6, 11, 12, 15, 17, 19, 20, 24, 25, 28, 29, 31, 32, 33, 36, 37, 38, 39, and 40 were missing. In females, types 1, 2, 6, 11, 14, 15, 17, 20, 21, 22, 24, 27, 29, 30, 31, 32, 33, 34, 35, 36, 37, 39, and 40 were missing.

The gender distribution of the ‘S’ types on the left side is also shown in Table 6. In males, types 1, 5, 6, 13, 16, 18, 19, 20, 23, 24, 25, 26, 27, 30, 34, 35, 37, and 38 were missing. In females, we did not identify types 4, 5, 11, 13, 15, 16, 17, 18, 21, 23, 26, 28, 30, 31, 32, 33, 34, 35, 36, 39, and 40.

The present study identified 40 different S-types of ECA branching sequences, demonstrating extraordinary anatomical variability in the origin and sequencing of the APA relative to other ECA branches. Despite observing 14 right-only and 9 left-only S-types, as well as 13 male-only and 8 female-only types, comprehensive statistical analysis revealed no significant side-related (χ^2^ = 42.12, df = 39, *p* = 0.379, Cramer’s V = 0.50) or gender-related (χ^2^ = 49.81, df = 39, *p* = 0.138, Cramer’s V = 0.54) differences in the overall distribution of S-types. Both comparisons showed medium effect sizes but failed to reach statistical significance, indicating substantial variability without systematic lateralization or sexual dimorphism.

Furthermore, S0 (absent or ICA-origin of APA) showed identical prevalence between sexes (19.4% in males vs. 19.4% in females, χ^2^ = 0.00, df = 1, *p* = 1.000, Cramer’s V = 0.00) and no significant side preference (16.5% right vs. 22.4% left, χ^2^ = 0.61, df = 1, *p* = 0.438, Cramer’s V = 0.14). The absence of effect for sex (V = 0.00) and minimal effect for side (V = 0.14) indicate that APA absence or ectopic ICA origin occurs randomly without demographic or laterality patterns.

Even when analyzing the most common S-types (types 7, 9, 8, 10, and 4) or grouping by anatomical features (presence of OPT, LFT, or TLT), no statistically significant patterns emerged (all *p* > 0.05). The observed side-specific and gender-specific types were predominantly rare variants (*n* = 1–2 cases), representing sampling variation rather than true biological lateralization or sexual dimorphism.

Multiple regression analysis revealed that the hyoid level of APA origin significantly predicted S-type classification on the right side (β = 5.71, SE = 1.48, t = 3.85, *p* < 0.001), while APA origin type (β = −0.52, SE = 0.57, t = −0.91, *p* = 0.365) and gender (β = −2.03, SE = 2.08, t = −0.97, *p* = 0.333) did not contribute significantly. The overall right-side model was statistically significant (R^2^ = 0.165, adjusted R^2^ = 0.134, F(3, 81) = 5.33, *p* = 0.002), explaining 16.5% of variance in S-type classification. The Durbin–Watson statistic (1.96) indicated no autocorrelation concerns.

Similarly, for the left side, hyoid type significantly influenced S-type classification (β = 6.17, SE = 1.90, t = 3.25, *p* = 0.002), while APA origin (β = 1.01, SE = 0.59, t = 1.71, *p* = 0.092) and gender (β = 0.35, SE = 2.29, t = 0.15, *p* = 0.880) showed no significant effects. The left-side model was also significant (R^2^ = 0.200, adjusted R^2^ = 0.170, F(3, 81) = 6.74, *p* < 0.001), explaining 20.0% of variance. The Durbin–Watson statistic (1.74) indicated no autocorrelation concerns.

### 3.6. Thyrolingual Trunks in the Investigated Lot

In 170 sides were found two cases with TLT, one with the S-type 33 (TLT from CCA, and APA-FA from ECA) on the left side, and the second with the S-type 35 (TLT-OA-APA-FA) on the right side. These will be further detailed.

#### 3.6.1. Thyrolingual Trunk Originating from the Common Carotid Artery

The ascending pharyngeal artery arises from the origin of the external carotid artery as its first branch (type 33). Contralateral long extracranial inferior petrosal sinus and ascending pharyngeal artery from the internal carotid artery.

On the right side (Figure 7 and Figure 8), a 4.23 cm long styloid process was found, with a medial inclination of 72.4°. It crossed anteriorly, above the transverse process of the atlas, two veins: laterally—the internal jugular (IJV) vein, which had a transverse diameter of 5.94 mm, and medially—a long inferior petrosal sinus (IPS), with a transverse diameter of 3.15 mm. The IJV continued laterally to the transverse process of the atlas, and the inferior petrosal sinus, anterior to it. It was observed that the compression of the IJV occurs between the transverse process of the atlas and the posterior aspect of the digastric muscle. Also, the IPS appeared compressed at this level. The IJV had a minimum diameter of 1.21 mm, and the IPS had a minimum diameter of 1.08 mm. The IPS crossed laterally the ICA and APA, which, in turn, originated from the ICA (type IV), and continued medial to the ECA, crossed the origin of the FA, and descended anterior to the ECA, over the initial loop of the LA. The IPS and ECA coursed on the lateral side of the greater hyoid horn. Inferior to the hyoid, the IPS crossed the STA laterally and continued obliquely posteroinferiorly over the CCA, emptying into the IJV at 3.59 cm inferior to the CB. The APA on that side arose from the anteromedial wall of the origin of the ICA, at the hyoid level.

On the left side (Figure 9), the APA originated 8.9 mm posterosuperior to the tip of the greater hyoid horn from the posterior wall of the origin of the ECA, as its first branch. This was because the LA and STA arose via a common TLT from the CCA. The TLT origin was 8.8 mm inferior to the greater hyoid horn; the length of this common arterial trunk was 5.1 mm. It divided at 5 mm lateral to the superior horn of the thyroid cartilage into two vertical branches—an upper one, the LA, which ascended over the greater hyoid horn and made an upper loop before continuing with the hyoglossal segment, and a second, inferior one, the STA.

#### 3.6.2. Thyrolingual Trunk Originating from the External Carotid Artery

In one case (Figure 10), we found on the right side the S-type 35 of APA: TLT-OA-APA-FA. On the left side, the S-type 36 was found to be absent, with STA, OA-LA-APA (with 2 roots), and FA.

The right CB was posterior to the hyoid tubercle. A 2.8 mm long TLT left the ECA immediately superolateral to the hyoid tubercle, 0.76 cm distal to the ECA’s origin. The STA thus descended over the hyoid tubercle and made a wide anterior loop, 1.53 cm anterior to the superior horn of the thyroid cartilage. The right STA then sent a right thyroid ramus to the right thyroid lobe and a left thyroid ramus to the left thyroid lobe, thus compensating for the absence of the opposite STA.

The hyoid bone was collapsed over the thyroid cartilage and tilted to the right side.

### 3.7. The S40 Type of the Origin of the External Carotid Artery’s Branches

In one of the studied cases, we found, on the left side, an unexpected arterial variant, corresponding to the type S40 (STA/APA from the CB, OA/SCM br.-LA). The CB was subgonial, 1.82 cm posterosuperior to the hyoid tubercle. From the medial aspect of the CB, the left two branches were as follows: one descending—the STA, and the other ascending—the APA (Figure 11). The ECA and ICA made an angle of 62.39°, opened superiorly. The initial segment of the ECA, 1.53 cm long, was directed anterosuperiorly, and then the ECA made an angle of 118.85°, opening posteriorly to further continue with the retromandibular course. At 6 mm distal to the origin of the STA and APA, the LA, OA, and a SCM branch originated from the ECA at the same level. The SCM branch originated from the lateral wall of the ECA, the AO from the inferomedial wall of the ECA, and the OA from the superolateral wall of the ECA. At 0.93 cm distally to the origin of the latter, the FA originated from the ECA.

The SCM branch traversed the venous fork between the common facial vein and the IJV, deep into the sternocleidomastoid.

The left STA had a 1.39 cm suprahyoid segment from which, 4 mm superior to the hyoid tubercle, the superior laryngeal artery arose. The latter described an ‘S’-shaped course, passing at 4.2 mm medial to the hyoid tubercle and then returning laterally, applied to the lateral aspect of the thyrohyoid membrane. The left ECA trifurcated into the maxillary, superficial temporal, and transverse facial arteries.

## 4. Discussion

The number of branches and branching pattern of the ECA are variable, impacting various applications, such as embolization, chemotherapy, cervical discectomy, and thyroid surgeries. The APA holds significant clinical importance in head and neck vascular pathology [22]. It frequently supplies various lesions, including dural arteriovenous fistulas, paragangliomas, direct arteriovenous fistulas between the APA and IJV, and contributes to epistaxis [22]. Beyond its pathological involvement, the APA serves dual therapeutic and compensatory functions: it provides a transarterial access route for endovascular treatment of these lesions and establishes crucial collateral circulation in cases of vessel occlusion or stenosis [22]. While APA aneurysms represent a rare occurrence, the vessel’s multifaceted role in both disease processes and compensatory mechanisms underscores its critical importance in neurovascular and head and neck surgery [22]. However, transarterial embolization procedures involving the APA carry substantial risks, including cranial nerve injuries, cerebellar infarction, and embolic agent migration, with reported permanent morbidity rates of 7.5% [14].

Variant APA origins fundamentally alter the risk profile across multiple clinical scenarios.

Risk during internal carotid artery exposure

An APA arising from the ICA or CB is often located on the dorsal or medial aspect, making it obscured and at higher risk of inadvertent injury during ICA exposure, especially in standard anterolateral approaches. Manipulation or cross-clamping in these cases increases the risk of cranial nerve injury and unexpected back-bleeding, particularly if the APA is not preoperatively identified [23,24,25].

Aberrant APA origins may also serve as collateral pathways in ICA occlusion, complicating exposure and increasing the risk of ischemic complications if damaged [26].

When the APA originates from the ICA (Type IV, 4.71%), it presents unexpectedly during vessel mobilization, risking inadvertent injury and potentially simulating stenotic ICA [5,23,27]. APA absence (14.71%) or variant origins from FA, LA, or LFT create additional surgical uncertainty [8,23,28].

Risk during carotid endarterectomy

Anomalous APA origins (from ICA or CB) can result in incomplete control of blood flow during carotid endarterectomy (CEA), risking intraoperative bleeding. Detection and clamping of the APA origin are essential, and rotation of the ICA within the carotid sheath may be required to visualize and control the vessel [23,24,25].

Preoperative 3D angiography is recommended to identify APA variants and reduce the risk of cranial nerve injury and hemorrhage [23,24].

Standard technique manages the APA indirectly through ECA clamping, but anomalous origins require direct manipulation. Akiyama et al. (2025) documented APA manipulation in 15.8% of carotid endarterectomies, with 5.70 times higher cranial nerve injury odds (*p* = 0.086) [23]. The mechanism involves proximity to cranial nerves IX-XII and the superior cervical ganglion [5,23,27]. ICA-origin APA causes persistent back-bleeding [23]. High CB increases manipulation requirements (32.4% vs. 13.5%, *p* = 0.005) [23]. OPT (17.06%) and duplicated APAs (1.4%) complicate control [23,29].

Endovascular access route modifications

Variant origins alter the endovascular approach. An APA from the ICA or bifurcation may be more challenging to catheterize and increases the risk of inadvertent entry or injury during interventions such as stenting or embolization [30].

Aberrant APA origins can provide rare collateral routes in ICA occlusion, affecting the direction and flow dynamics during endovascular procedures [26].

For standard ECA-origin APA (Types I and II, 62.35%), interventionalists advance a guide catheter to the ECA origin, then navigate a microcatheter selectively into the APA orifice. The S-type classification guides this navigation; for example, in S-type 7 (STA-LFT-APA-OA, 7.1% prevalence), selective APA catheterization requires advancing past the large LFT ostium to avoid non-target embolization [5]. The OPT origin (17.06%) demands superselective navigation into the APA component rather than the occipital component [5]. ICA-origin APA (4.71%) fundamentally alters the endovascular approach, requiring navigation through the internal carotid system with substantially higher thromboembolic risk [5]. Variant origins from the FA, LA, or LFT (Type V, 1.18%) require selective catheterization of these vessels first, which is technically challenging given their tortuous courses [8,28]. Duplicated APAs require embolization of both vessels to achieve complete devascularization [23,29].

Danger of inadvertent embolization

The APA supplies critical neural and nasopharyngeal structures. Variant origins, especially from the ICA, increase the risk of non-target embolization, potentially leading to cranial nerve deficits or brainstem ischemia if embolic material refluxes into the ICA or its branches [5,30].

Detailed anatomical assessment and careful angiographic visualization are crucial to avoid inadvertent embolisation, particularly when the APA arises near the ICA or bifurcation [30].

The APA’s meningeal trunk supplies cranial nerves IX-XII and maintains vertebrobasilar collaterals [5]. Embolization carries 7.5% permanent morbidity from cranial nerve palsies [5]. Anastomoses to the vertebral artery through hypoglossal, muscular, or odontoid branches enable embolic material migration, risking cerebellar infarction or posterior circulation stroke [5]. An ICA origin of the APA creates high-risk intracranial anastomoses through carotid ramus, clival branches, or tympanic connections, potentially causing ophthalmoplegia [8,28], pituitary dysfunction, or brainstem infarction [5]. OPT embolization (17.06%) risks posterior scalp necrosis [5]. LFT variants risk facial and tongue embolization via maxillary artery connections [5,28]. Preoperative CT/MR angiography identifying APA variants is essential [23,29]. Our 40 distinct S-types emphasize individualized anatomical assessment.

### 4.1. On the Anatomic Variations in the Ascending Pharyngeal Artery

#### 4.1.1. Preexisting Studies of the Ascending Pharyngeal Artery

Various previous studies have assessed different possible origins of the APA (Table 7), from the OA, ICA, CCA, CB, FA, LA, LFT, or from the ascending cervical artery [1,5,25,27,31,32,33,34,35]. Both the APA and OA may originate from the ICA [36].

Bergman et al. (1988) listed in their *Compendium of human anatomic variation* the possibilities of origin of the APA: from the ECA or the CB (65–80%), the OA (14–20%), or the CCA (7–9%) [42]. They also noted that the ECA may provide an accessory APA [42]. Lippert and Pabst (1985) documented that the APA originates directly from the ECA in 70% of cases, from the OA (OPT) in 20% of cases, from the FA in 2% of cases, and from the ICA in 8% of cases [8].

Lasjaunias and Moret (1976) reported a 65% prevalence of ECA origin for the APA, citing Poirier (1895), not from their own case series [43,44]. However, they presented a case in which the ascending palatine artery and APA left from a common trunk, but unfortunately, the authors did not specify the parent vessel of this common trunk in their description [44]. Li et al. (2024) dissected 20 sides in 10 cadavers, but they just assumed the APA originated from the ECA in all cases and did not document any variations in its origin [2].

Only recently, evidence of a double APA was brought [29], and a lingual-facial-ascending pharyngeal trunk was reported [28].

Table 8 highlights that the analyzed study provides the most detailed breakdown of ECA wall origins (medial vs. posterior) and documents one of the highest APA absence rates (14.71%) among studies.

#### 4.1.2. The Absence of the Ascending Pharyngeal Artery

While explicit reports of complete congenital absence are lacking, failure to identify the APA may be due to anatomical variation, hypoplasia, or technical limitations in imaging [5,25]. The present study documented an absent APA (Type 0) prevalence of 14.71% (25 of 170 sides), representing one of the highest rates reported in the anatomical literature. This finding stands in marked contrast to the recently published data of Akiyama et al. (2025), who reported absent APAs in only 1.4% of 277 carotid sides examined by three-dimensional rotational angiography in patients undergoing CEA [23]. However, our results align more closely with the anatomical study of Cavalcanti et al. (2009), who documented absent APAs in 20% of cases (4 of 20 cadaveric sides) [5]. The substantial discrepancy between our findings and those of Akiyama et al. may reflect several methodological differences: first, our study employed angio-CT imaging in a general patient population rather than CEA candidates, potentially capturing a broader spectrum of anatomical variation; second, the definition and identification criteria for “absent” APAs may differ between radiological techniques, with smaller-caliber APAs potentially being undetectable on three-dimensional rotational angiography but visible on angio-CT; third, Akiyama’s study population consisted exclusively of patients with atherosclerotic disease requiring CEA, a pathological condition that might influence vascular anatomy through adaptive remodeling or collateral development. The convergence between our data (14.71%) and Cavalcanti’s findings (20%), despite different imaging modalities (angio-CT versus cadaveric dissection), suggests that the true prevalence of absent or angiographically undetectable APAs in the general population may be substantially higher than previously recognized from surgical series. This higher prevalence has important clinical implications for carotid surgery, skull base procedures, and embolization protocols, as the absence of a typical APA from the ECA may necessitate alternative collateral pathways through the ICA, vertebral system, or contralateral circulation. Our larger sample size (170 sides versus 20 in Cavalcanti’s study) provides more robust prevalence estimates while confirming the anatomical observation that APA absence, whether developmental or functional, represents a clinically significant anatomical variant rather than an exceptional rarity.

#### 4.1.3. The Double Ascending Pharyngeal Artery

Two different recent studies found double/duplicate APAs [23,29] with different approaches. Table 9 provides various parameters for direct comparison, making it easy to see how these two crucial 2025 studies complement each other in understanding APA duplication.

The studies of Akiyama et al. (2025) [23] and Calotă et al. (2025) [29] differ in four key aspects. First, study design: Akiyama’s prospective series provides prevalence data (1.4%), while Calotă’s case report offers detailed anatomical characterization. Second, classification approach: Calotă distinguishes true duplication from two-rooted variants, while Akiyama uses general “duplication” terminology. Third, anatomical detail: Calotă provides extensive morphometric data and hyoid bone relationships, whereas Akiyama focuses on origin patterns and surgical relevance. Fourth, clinical emphasis: Akiyama addresses CEA outcomes and CNI risk, while Calotă focuses on surgical anatomy and bleeding prevention.

#### 4.1.4. The Occipitopharyngeal Trunk

The OPT represents a common anatomical variation in which the APA originates from the OA rather than directly from the ECA. In our study, the OPT was identified in 29 of 170 sides (17.06%), with a notable side asymmetry: 14.12% (12/85) on the right side and 20% (17/85) on the left side. These findings align closely with classical anatomical literature, where Lippert and Pabst (1985) reported OPT prevalence of 14–20% and Bergman et al. (1988) documented OPT formation in 14–20% of cases [8,42]. The vertical topography of the OPT origin relative to the hyoid bone showed predominantly suprahyoid origins (14.71% overall), with rare hyoid-level (1.18%) and infrahyoid (1.18%) origins. In all identified cases, the OPT originated from the ECA. The presence of OPT was integrated within seven distinct S-type sequential patterns (types 6, 9, 10, 17, 20, 25, and 37), with type 9 (STA-LA-OPT-FA) being the most frequent configuration, observed in 6.47% of all sides. Type 10 (STA-OPT-LA-FA) showed a prevalence of 5.29%, while the remaining OPT-associated types were considerably rarer. Previous anatomical studies documented the OPT as an isolated variation. In contrast, our sequential classification system reveals that OPT occurs within specific branching patterns. The OPT’s position within the ECA sequence varies substantially across different S-types. This demonstrates the importance of documenting complete sequential anatomy rather than individual trunk variations alone.

#### 4.1.5. The Thyrolingual Trunk

We found two cases of this rare variant, the TLT, in our study. In the first one (type S 33), a left TLT originating from the CCA was found 8.8 mm below the greater hyoid horn, measuring 5.1 mm. It divided into the LA and STA. The APA was thus the first branch of the ECA. In the second case (type S 35), a right TLT long of 2.8 mm originated from the ECA immediately superolateral to the hyoid tubercle, within the sequence TLT-OA-APA-FA. The left side showed the absence of the STA.

Lemaire et al. (2001) reported a case of dissection with a 5.2 mm long TLT arising from the CCA [45]. Its origin was at the level of the upper pole of the thyroid lobe [45]. These authors documented 26 previously reported cases of TLT [45].

Lemaire et al. (2001) document a series of old publications in which 26 cases with TLT [45] were reported by: Adachi (1928) [46], Faller and Schärer (1947) [47], Livini (1900) [48], Poisel and Golth (1974) [49], Quain (1844) [50], Rogers (1929) [51], Sébileau and Gouverneur (1920) [52] and Vuillième and Bruneton (1932) [53], respectively. Although numerous, only in Vuillième and Bruneton’s 1932 study did a 10 mm long TLT leave the CCA [53]. In the case reported by Vuillième and Bruneton (1932), the superior laryngeal artery arose directly from the CCA [53]. Still, in the case reported by Lemaire et al. (2001), the superior laryngeal artery arose from the STA [45].

Vazquez et al. (2009) performed a study on 330 unilateral cervical specimens by dissection [54]. It is evident that this batch did not facilitate a bilateral appreciation of the variations in the branches of the ECA. The authors found in one specimen a TLT originating from the left ECA (0.6%) and in another specimen a TLT originating from the CCA (0.6%) [54]. As documented by Vazquez et al. (2009) [54], Quain (1844) found a TLT in 1/302 cases [50], Livini (1903) found TLTs in 3/200 cases [40], Poynter (1922) found one TLT in 200 cases [55], Aaron and Chawaf (1967) found TLTs in 11/187 cases [56], and Poisel and Golth (1974) found TLTs in 5/156 cases [49].

Cobiella et al. (2021) performed a study on 193 cadavers and found TLTs in just 1.04% (2/193) [33]. The two TLTs found by these authors were both on the right side, one with origin from the CCA and the other with origin from the ECA [33]. Cobiella et al. (2021) did not describe the anatomical variants contralateral to the variants recorded in their study [33], as we did here. Natsis et al. (2011) studied 100 carotid arteries in cadavers and found 3 TLTs (3%), one from the ECA, one from the CB, and the third from the CCA [57]. It is evident that such a carotid axes batch did not allow bilateral appreciation of the variations in the ECA branches. Iwai et al. (2012) reported a case with TLT from the CCA [58], but these authors also did not document contralateral jugulocarotid anatomy. Nochikattil et al. (2017) reported a case in which a TLT was found intraoperatively originating from the CCA [59]. This report was not subsequently documented by Tsakotos et al. (2024), who reported a similar variant [60]. Being an intraoperatively discovered variant, Nochikattil et al. (2017) [59] did not document the bilateral morphology of the ECA nor the ipsilateral S-types with the reported variant. Herrera-Nunez et al. (2020) found TLTs in 16/152 cases (10.5%) [61] but did not show the sequence of the ipsilateral branches of the ECA and did not bilaterally assess the ECA morphology. Tsakotos et al. (2024) reported a case of TLT with CCA origin, documented by angio-CT [60]. The authors also present the morphology of the contralateral ECA, but did not specify the exact sequence of the origin of the branches of the two ECAs [60]. A TLT with origin from the right CEA was recently reported in a series of cases, with the hyoid collapsed over the thyroid cartilage of the larynx [21]. The sequencing of the branches of the two ECAs has not been shown in detail in this report either.

Therefore, the present study provides the first bilateral documentation of TLT variants with complete contralateral morphology and the first detailed S-type sequencing for TLT variants (types 33 and 35). Novel precise measurements include TLT lengths (5.1 mm and 2.8 mm), division points, and specific anatomical relationships to landmarks such as the greater hyoid horn and superior horn of the thyroid cartilage. Unique associated findings were documented, including the STA compensation pattern where the right STA supplied both thyroid lobes in the absence of the contralateral STA, a long extracranial inferior petrosal sinus, and hyoid bone position anomalies. The comprehensive bilateral analysis of all 170 sides from 85 individuals allowed for complete characterization of ipsilateral branch sequences and contralateral variations, a methodological approach not employed in previous TLT case reports [21,33,45,54,57,58,59,60,61].

#### 4.1.6. The S40 Variant of ECA Branching

The S40 variant we found represents a rare branching pattern of the ECA, characterized by the simultaneous origin of the STA and APA directly from the CB. This configuration deviates significantly from the typical sequential branching pattern, in which these vessels arise independently of the ECA trunk.

The clustering of the LA, OA, and SCM branches at a single level creates a complex anatomical landmark that surgeons must recognize during neck procedures. The proximity of these vessels to the CB (6 mm distally) reduces the available working space for surgical manipulation.

The SCM branch traversing the venous fork poses a particular risk during IJV or lymph node dissection. Additionally, the unusual “S”-shaped course of the superior laryngeal artery, which passes medially then returns laterally to the hyoid, could complicate thyroidectomy or laryngeal procedures. Recognition of such variants is essential for preoperative imaging interpretation and surgical planning to prevent inadvertent vascular injury.

#### 4.1.7. The Sternocleidomastoid Branch of the Proximal Segment of the External Carotid Artery

Different arteries can give SCM branches: ECA, STA, FA, OA, LA, and the suprascapular artery [3,48,49,50,51,52,53,62]. Sternocleidomastoid branches have also been identified from the transverse cervical artery, thyrocervical trunk, and superficial cervical artery [63].

Direct branches from the ECA vascularize the middle third of the SCM muscle with different prevalences; on the right/left—in 27%/20% of cases, only by branches from the ECA, and in 20%/27% by branches from both the ECA and the STA [63]. These prevalences were estimated differently by the authors. Thus, another study found in the middle third of the SCM muscle only branches from the ECA in 23% of cases on both sides, and the ECA and STA vascularized the middle third of the muscle equally in 27%/26% on the right/left [64]. In one case, the authors found the middle third of the SCM muscle vascularized from the LA and ECA but not from the STA [64]. Also, the authors found in 12 specimens (31 cadavers, not specified whether uni- or bilateral) a particular course of the SCM branch of the ECA: it had an initially ascending course, wrapped around the hypoglossal loop and then descended to the level of the anterior margin of the SCM muscle about 2 cm, entering the muscle at the junction of the upper and middle thirds [64].

Froes et al. (1999) studied only the SCM branches of the AO by latex injections [65]. They revealed a deep, longitudinal, descending SCM branch of the OA (the superior arterial pedicle of the SCM muscle) that descended parallel to the longitudinal axis of the muscle and sent transverse twigs [65]. One such deep longitudinal ramus was also evidenced here, but from the ECA—it descended deep to the SCM muscle, after describing a superior loop lateral to the ECA and OA, giving the false appearance of a long ramus of the OA.

The SCM flap can be used to cover defects of the buccal floor, pharynx, and mandible, as well as defects resulting from parotidectomy or due to Frey syndrome [63]. This flap is helpful because the SCM muscle has a rich vascularization; it can be used as a flap in combinations, the two ends of the muscle can be moved, and the esthetic results are good [63]. However, this flap is unpopular among head and neck surgeons [63].

#### 4.1.8. The Sequence of Origins of the External Carotid Artery’s Branches

Our identification of 40 distinct S-types represents unprecedented documentation of the extraordinary anatomical diversity of the external carotid arterial system, thus a significant original contribution to the anatomical literature.

To our knowledge, this is the first study to provide a comprehensive sequential classification system for ECA branching patterns, integrating the APA’s position within the complete ECA branch sequence. While numerous previous studies have documented individual trunk variations, including LFT with prevalences of 17–20% [33,66,67,68], TLT at 1–10.5% [57,60,61], and OPT at 14–20% [8], none have systematically recorded the complete sequential order of all ECA branches with bilateral assessment. Existing classification systems have been limited in scope. Yamamoto et al. (2019) categorized ECA variations into types A, B, and C based solely on the number of simultaneously arising branches (2, 3, or 4+), without documenting sequential order [69]. Cobiella et al. (2021) proposed a dual system with patterns I–IV for anterior branches and types a–d for posterior branches [33]. However, they did not specify the exact branch sequence or assess bilateral patterns. Multiple case reports documenting TLT variations consistently omitted complete ipsilateral branching sequences and contralateral anatomy [57,58,59,60,61]. Our study identified 40 distinct S-types through bilateral angio-CT assessment in 85 patients (170 sides). This represents a qualitative advance beyond previous partial classifications through four key features. First, we documented the complete sequential order of all ECA collateral branches. Second, we integrated trunk variations (LFT, TLT, OPT) within their sequential context. Third, we accounted for rare APA origins from the ICA, CB, or LA. Fourth, we systematically assessed bilateral patterns. This comprehensive approach revealed extraordinary anatomical diversity that cannot be captured by previous simplified systems. With 23 types present in fewer than 5 cases each, the findings emphasize the individualized nature of ECA branching morphology. The clinical utility of the S-type system lies in providing a complete anatomical roadmap rather than isolated information about individual structures. This comprehensive knowledge is particularly valuable for complex surgical planning, endovascular procedures, and preoperative risk assessment, where understanding the entire sequential pattern may be critical for avoiding iatrogenic complications.

No statistically significant side-related or gender-related differences were found in S-type distribution. The absence of statistically significant side-related or gender-related differences in S-type distribution has important embryological implications. The diversity of ECA branching sequences reflects developmental stochasticity in arterial morphogenesis rather than genetically determined patterning. During the 4th to 8th gestational weeks, the external carotid system forms through selective persistence and regression of ventral pharyngeal arteries, with the APA specifically representing a cranial extension of the dorsal aortic root [15]. The sequential branching patterns documented in this study (40 distinct S-types) arise from variable timing of pharyngeal arch artery sprouting, differential regression of embryonic vascular channels, and persistence of normally transient anastomoses between adjacent arterial territories [18]. The lack of systematic lateralization suggests that hemodynamic forces during embryogenesis, rather than genetic asymmetry programs, predominantly influence final vascular configurations [16]. Similarly, the absence of sexual dimorphism in branching patterns indicates that sex hormones do not significantly modulate pharyngeal arch artery development. The observation that Type 0 (absent APA) occurs in 14.71% of cases may represent complete regression of the dorsal aortic root cranial extension, while Type IV (ICA origin, 4.71%) likely reflects persistence of an embryonic connection between the APA anlage and the third aortic arch derivative (ICA precursor) that normally disappears during development [15]. The formation of common trunks (OPT in 17.06%, LFT, TLT) reflects incomplete separation of adjacent pharyngeal artery sprouts or persistence of shared embryonic origins from the ventral aorta. Our finding of double APA [29] can be explained by the separate insertion and incomplete fusion of middle and inferior pharyngeal arterial branches during morphogenesis [18]. This embryological framework (Table 10) explains why individual anatomical variation is the norm, why bilateral symmetry cannot be assumed, and why preoperative imaging is essential for surgical planning in the carotid region. Rare embryological anomalies such as persisting carotid duct connections between the third and fourth aortic arches can result in proximal ECA agenesis and atypical reconstruction of the arterial supply [70], demonstrating the potential for extreme variations in carotid system development.

The absence of statistically significant side-related or gender-related patterns in APA origin sequences has important implications for clinical practice and surgical planning. Clinicians cannot reliably predict contralateral carotid anatomy based on ipsilateral findings, nor can patient gender be used to anticipate specific branching patterns or the presence of arterial trunks such as OPT or TLT. This underscores the critical importance of bilateral imaging assessment in preoperative planning for procedures involving the carotid bifurcation, skull base approaches, or interventions in the parapharyngeal space. The identification of 40 distinct S-types, many representing rare variants, underscores the need for individualized anatomical evaluation rather than relying on “typical” or “standard” vascular anatomy. For endovascular procedures, embolization protocols, head and neck tumor surgery, and carotid endarterectomy, preoperative CT or MR angiography should be performed bilaterally to map the specific branching sequence and identify potential variations such as APA origin from the ICA (type IV, 4.71%), OPTs (17.06%), or rare trunks including TLTs. The unpredictability of contralateral anatomy also has implications for surgical teaching and anatomical education, highlighting that anatomical variation is the norm rather than the exception in this region, and that comprehensive imaging remains the gold standard for understanding an individual patient’s vascular architecture.

#### 4.1.9. Clinical Applications of the S-Type Classification System

The S-type sequential classification system provides surgeons with a comprehensive anatomical roadmap that enhances procedural safety and surgical planning across multiple clinical scenarios. The following examples illustrate specific applications:

Carotid endarterectomy (CEA)

During CEA, the sequential branching pattern directly impacts arterial clamping strategy and bleeding control. Consider a patient presenting with S-type 21 (STA-APA-LA-FA-OA): the APA arises as the second branch from the ECA, positioned between the STA and LA. According to Akiyama et al. (2025), APA manipulation, including cross-clamping, was required in 15.8% of CEA cases when the APA originated within the designated arterial clamping site [23]. In S-type 21, preoperative knowledge allows surgeons to anticipate that the APA will be encountered early in the proximal ECA dissection, requiring additional exposure and potential cross-clamping to prevent unexpected back-bleeding during arteriotomy.

Contrast this with S-type 4 (STA-LA-APA-OA-FA), where the APA emerges as the third branch, positioned more distally. This configuration allows standard clamping distal to the LA origin without encountering the APA, simplifying the procedure. The critical distinction is not merely knowing that an APA exists, but understanding its exact sequential position relative to the surgical field boundaries.

For S-type 9 and S-type 10 (both involving OPT), the challenge intensifies. In S-type 9 (STA-LA-OPT-FA), the OPT arises as the third branch, representing a single vessel that must be controlled to prevent back-bleeding from both the OA and APA. Failure to recognize this configuration could result in inadequate hemostasis despite apparently controlling all visible vessels. As Akiyama et al. documented, complete control of back-bleeding was achieved in all 279 CEA cases when surgeons had preoperative 3D angiographic knowledge of branching patterns [23].

The S40 pattern (STA/APA from CB-OA/SCM br.-LA-FA) presents a unique surgical challenge: both the STA and APA originate directly from the CB rather than the ECA. During CEA, this means the APA cannot be controlled by standard ECA clamping and requires separate attention at the bifurcation level itself. Without preoperative recognition, surgeons might be surprised by persistent bleeding from an APA they assumed would be controlled by proximal clamp placement.

Skull base surgery and parapharyngeal space approaches

For transcervical approaches to the skull base or parapharyngeal space tumors, the S-type classification predicts the vascular anatomy encountered during deep dissection. Consider S-type 14 (STA-APA-OA-LA-FA): here, the APA is the second branch and will be encountered early and superficially during lateral neck dissection before reaching the LA and FA. This sequence indicates that APA ligation (if necessary for tumor devascularization) can be performed without disturbing the more distal LA and FA supply to the tongue and face.

In contrast, S-type 38 (STA-LA-OA-APA-FA) positions the APA as the fourth branch, deep within the dissection field. Accessing this vessel requires working past the LA and OA, with increased risk of injuring these anterior vessels during APA identification. For tumor embolization planning, surgeons must know whether the APA will be accessible early (proximal sequences like S-types 13, 14, 21, 23) or require deeper dissection (distal sequences like S-types 12, 26, 38).

The presence of Type IV APA (ICA origin, 4.71%), classified as S0, fundamentally alters the surgical approach. In standard skull base procedures, surgeons expect to find the APA arising from the ECA medial wall. When the APA actually originates from the ICA, it courses in an entirely different trajectory, running along the ICA rather than the ECA territory. This variant was identified in cases that might otherwise have been classified as “absent APA” if only the standard ECA territory had been examined. Recognition prevents inadvertent injury during ICA manipulation.

Endovascular Embolization Procedures

For preoperative tumor embolization (such as juvenile nasopharyngeal angiofibromas or paragangliomas), the S-type sequence guides the catheter navigation strategy. In S-type 7 (STA-LFT-APA-OA), the most common type (7.1% prevalence), an LFT precedes the APA. This means selective catheterization of the APA requires advancing past the large LFT ostium, with careful microcatheter technique to avoid inadvertently entering the facial or lingual circulation.

When an OPT is present (S-types 6, 9, 10, 17, 20, 25, 37), embolization becomes more complex. For S-type 10 (STA-OPT-LA-FA, 5.9% prevalence), the OPT is the second branch from the ECA. Superselective catheterization into the APA component of the OPT (rather than the OA component) requires understanding the trunk’s internal bifurcation geometry. Embolic material injected too proximally will affect both the APA and OA territories, potentially causing unwanted devascularization of the posterior scalp or inadvertent cranial nerve ischemia.

The sequential classification also predicts collateral pathways during vessel occlusion. In S-type 40, where the APA arises from the CB alongside the STA, it likely has reduced anastomotic connections with adjacent ECA branches compared to standard ECA-origin APAs. This affects decisions about proximal versus distal embolization and influences the risk of non-target embolization through collateral channels.

Selective Intra-Arterial Chemotherapy

For head and neck cancer treatment with intra-arterial chemotherapy, drug delivery depends on precise vascular mapping. Consider a patient with an oropharyngeal tumor supplied partly by the APA. If the patient has S-type 2 (STA-LA-APA/OA-FA) with simultaneous APA and OA origin, catheter positioning must account for this shared ostium. Drug infusion here will perfuse both APA and OA territories simultaneously, affecting both the tumor (via APA) and the posterior scalp (via OA).

Compare this to S-type 4 (STA-LA-APA-OA-FA), where the APA and OA have separate origins. Here, truly selective APA infusion is possible without OA contamination, allowing higher local drug concentrations to the tumor while sparing the scalp and occipital muscle territories. The sequential classification thus directly impacts therapeutic selectivity and dosing calculations.

For tumors with bilateral vascular supply, the observation that S-types show no systematic lateralization (χ^2^ = 42.12, *p* = 0.379) has important implications: surgeons cannot predict the right-side S-type from knowledge of the left-side pattern. Bilateral selective catheterization may require entirely different navigation strategies for each side, even in the same patient during the same procedure.

Emergency Vascular Control and Trauma Surgery

In trauma scenarios involving penetrating neck injuries with active hemorrhage, rapid vascular control depends on predicting which vessels may be injured based on the injury trajectory. The S-type classification allows trauma surgeons to anticipate the sequential vascular anatomy encountered during emergency neck exploration.

For an injury in the upper carotid triangle (near the hyoid level), knowing the hyoid-related topography combined with S-type sequencing predicts whether an APA injury is likely. In cases with infrahyoid APA origins (IH type, 8.24% prevalence), the APA will be encountered lower in the neck than expected, potentially explaining persistent bleeding despite controlling more typical injury locations.

The identification of double APA (as documented in Calotă et al., 2025) [29] demonstrates that controlling one APA does not ensure complete hemorrhage control. The S-type system, by emphasizing complete sequential documentation, reminds surgeons to actively search for all potential variant vessels rather than assuming anatomy based on the first structure identified.

Preoperative planning integration

The practical application of S-types requires integration into preoperative imaging protocols. Based on our findings, (a) bilateral imaging is mandatory because with no predictive value from contralateral anatomy (*p* = 0.379), both sides must be independently characterized. (b) Radiologists should report not just the presence of trunks (OPT, LFT, TLT) but the complete branching sequence, allowing assignment of an S-type. (c) Certain patterns warrant special surgical attention: S40 (CB origin of STA/APA) requires bifurcation-level vascular control, S0 (Type IV, ICA origin of the APA) necessitates ICA-focused dissection rather than ECA territory, in OPT-containing S-types (6, 9, 10, 17, 20, 25, 37), a single vessel controls two arterial territories, infrahyoid S-types are a suggest for vessels located lower than typical external landmarks, and (d) in rare variant alerting, with 23 S-types present in <5 cases each (57.5% of all types), the default assumption should be variation, not “textbook” anatomy.

Surgical Teaching Applications

For anatomical education and surgical training, the S-type system provides several advantages. Residents can review the S-type distribution to understand which patterns are common locally and which are rare. This creates realistic expectations for surgical training. When intraoperative bleeding or unexpected anatomy occurs, retrospective S-type assignment helps determine whether the issue stemmed from rare anatomy (e.g., S40, S0) or from inadequate preoperative assessment of more common variants. Surgical simulators can incorporate S-type variation, ensuring trainees experience diverse anatomy during skills acquisition rather than only “standard” configurations. The key pedagogical insight from the present study is that with 40 distinct S-types and no predictive patterns by side or sex, anatomical variation IS the standard. Training paradigms that emphasize a single “normal” branching pattern inadequately prepare surgeons for the diversity they will encounter in practice.

#### 4.1.10. Diagnostic Pitfalls in Vascular Patients Caused by Anatomical Variants of the Ascending Pharyngeal Artery

Such diagnostic pitfalls could be classified as follows: (1) misclassification of ICA occlusions: the operable short-segment of ICA occlusions mistakenly classified as inoperable; (2) vessel misidentification: the APA confused with the ICA itself; (3) false dissection diagnosis: variant APA mistaken for pathological double lumen in arterial dissections; (4) non-visualization on imaging: APA not detected on preoperative 3D rotational angiography despite being present; (5) occlusion versus variant origin: inability to distinguish between APA occluded by extensive carotid plaque versus anomalous origin from the ascending cervical artery; (6) detection failure: small vessel caliber (approximately 1 mm diameter) escaping perception on standard imaging modalities.

Anatomical variants of the ascending pharyngeal artery pose significant challenges in cerebrovascular imaging, particularly during carotid duplex ultrasound examinations [39]. When the APA originates anomalously from the internal carotid artery rather than its typical external carotid origin, sonographers and clinicians may commit critical diagnostic errors with serious therapeutic implications [39].

The most significant pitfall involves misclassifying surgically treatable ICA occlusions as inoperable lesions, potentially denying patients beneficial interventions [39]. Furthermore, mistaking the variant APA for the ICA itself can lead to fundamentally flawed anatomical interpretation [37], while confusion with pathological double lumen may prompt unnecessary workup for arterial dissection [38,39]. These misinterpretations can lead to inappropriate treatment choices or missed surgical opportunities [39].

Recognition of these variants demands meticulous attention to vessel caliber, flow patterns, and parallel vessel courses during duplex sonography [39]. During carotid endarterectomy, failure to identify anomalous APA origins may result in unexpected back-bleeding or inadvertent vessel injury [23]. In approximately 0.7% of cases, the APA may not be visualized on preoperative 3D rotational angiography, either due to occlusion by extensive carotid plaque or anomalous origin from the ascending cervical artery [23].

The lack of dedicated imaging protocols, specifically using CT or MR angiography, that focus on APA anatomy further compounds these challenges. Recent case reports of duplicated and two-rooted APAs emphasize that identifying one APA does not necessarily exclude a distal vessel, warranting additional caution during open-field surgeries [29]. Heightened awareness of APA anomalies is therefore essential for accurate cerebrovascular diagnostics and optimal patient management [39].

### 4.2. On the Terminal Trifurcation of the External Carotid Artery

We found in the case with the S40 type of sequence of the ECA’s branches, a terminal trifurcation of the ECA into the maxillary, superficial temporal, and transverse facial arteries. Terminal branching variations of the ECA (Table 11) present significant challenges during parotid surgery, infratemporal fossa approaches, and superficial temporal artery procedures [71,72]. Terminal trifurcations and the exceptionally rare pentafurcations expose multiple arterial branches at a single anatomical point, substantially increasing hemorrhage risk during surgical dissection [72]. When the middle meningeal artery arises directly from the ECA rather than from the maxillary artery’s mandibular segment, neurosurgical approaches to the middle meningeal artery and infratemporal fossa require modified techniques [71]. Variations in superficial temporal artery branching, including absent frontal or parietal branches (occurring in approximately 3% of cases), or additional branches, directly impact temporal artery biopsy adequacy, scalp reconstruction, and bypass procedures [73]. Preoperative CT or MR angiography is recommended before complex head and neck procedures to identify these variants, enabling surgeons to modify approaches, minimize operative time, and reduce vascular injury risk [71].

### 4.3. On the Long Inferior Petrosal Sinus

The IPS is a paired dural venous sinus in the posterior cranial fossa that drains the cavernous sinus into the jugular bulb, receiving inflow from auditory structures and the brainstem [77,78]. Endovascular access to the IPS has diagnostic and therapeutic utility for conditions involving the cavernous sinus and sellar regions, including embolization of cavernous dural arteriovenous fistulas and venous plexuses of the skull base [77,79]. Bilateral IPS sampling is essential for diagnosing and differentially diagnosing pituitary microadenomas [79]. A long extracranial IPS courses along the IJV to empty at a lower level [79], functioning as an accessory IJV [80]. A long IPS may be compressed along with the IJV between the styloid process and the atlas [81]. We report here a case of such compression that was observed in a patient with a TLT variant.

### 4.4. Study Limitations

Several limitations should be considered when interpreting these findings. First, the retrospective observational design introduces selection bias, as patients underwent angio-CT for specific clinical indications rather than representing a random population sample. Second, the sample size of 85 patients (170 carotid axes) provided adequate power for common variants but limited characterization of rare patterns; 23 S-types occurred in fewer than five cases each. Third, imaging resolution constraints may not detect vessels < 1 mm in diameter, potentially contributing to the 14.71% APA absence rate. Advanced modalities such as catheter-based 3D rotational angiography might detect additional small vessels. Fourth, lack of systematic correlation with clinical outcomes limits validation of the classification’s clinical utility; prospective studies linking S-types to surgical complications or diagnostic accuracy are needed. Fifth, the single-center Romanian cohort limits geographic and ethnic generalizability. Finally, the complexity of 40 distinct S-types may challenge routine clinical application without simplified grouping strategies or standardized reporting templates.

Despite these limitations, this study represents the most comprehensive bilateral assessment of ECA branching sequences to date, providing a foundation for future prospective, multicenter investigations with larger sample sizes and clinical outcome correlation.

### 4.5. Future Research

Prospective multicenter studies should validate S-type distribution and correlate patterns with clinical outcomes (cranial nerve injury, catheterization difficulty, diagnostic accuracy). Advanced imaging must clarify whether APA absence (14.71%) represents true anatomical absence or imaging limitations. Artificial intelligence algorithms would enable automated classification and real-time surgical assistance. Simplified risk-based grouping systems would facilitate clinical adoption. Focused studies should establish the prevalence and management of rare variants (duplicated APA 1.4%, ICA origin of the APA 4.71%). Integration into electronic health records with standardized coding would enable outcome tracking. These investigations would transform S-type classification into a clinically actionable risk stratification tool.

## 5. Conclusions

This study presents the first comprehensive sequential classification system for ECA branching patterns, identifying 40 distinct S-types through bilateral angio-CT assessment. The APA exhibited remarkable origin variability: absent or ICA origin (14.71%), ECA medial wall (26.47%), ECA posterior wall (35.88%), occipitopharyngeal trunk (17.06%), ICA origin (4.71%), and other origins (1.18%). The OPT prevalence aligns with classical literature and occurs within seven distinct sequential patterns.

Statistical analysis revealed no significant side-related (*p* = 0.379) or gender-related (*p* = 0.138) differences in S-type distribution, with 23 types present in fewer than 5 cases each, underscoring the fundamentally individualized nature of carotid arterial anatomy. Clinicians cannot reliably predict contralateral anatomy based on ipsilateral findings, nor can patient sex reliably predict specific branching patterns.

For endovascular procedures, embolization protocols, head and neck surgery, and skull base approaches, bilateral preoperative CT or MR angiography is essential to map specific branching sequences and identify variations. The S-type system provides a comprehensive anatomical roadmap, extending beyond isolated trunk information, which is critical for complex surgical planning and the avoidance of intraoperative complications. Anatomical variation is the norm rather than the exception, making comprehensive bilateral imaging the gold standard for understanding individual vascular architecture.

## Figures and Tables

**Table 2:** Distribution of types 0–V (count, prevalence) of the origin of the ascending pharyngeal artery by side and gender. Abbreviations: R = right side; L = left side; Type 0 = absent APA; Type I = APA from ECA medial wall (ECA-M); Type II = APA from ECA posterior wall (ECA-P); Type III = APA from occipitopharyngeal trunk (OPT); Type IV = APA from internal carotid artery (ICA); Type V = APA from other origins (lingual artery, carotid bifurcation).

Type	0		I		II		III		IV		V	
Side	R	L	R	L	R	L	R	L	R	L	R	L
**males (108)**	8	8	18	8	16	27	7	10	5	-	-	1
	14.8%	14.8%	33.3%	14.8%	29.6%	50%	13.0%	18.5%	9.3%			1.9%
**females (62)**	3	6	14	5	5	13	5	7	3	-	1	-
	9.7%	19.4%	45.2%	16.1%	16.1%	41.9%	16.1%	22.6%	9.7%		3.2%	

**Table 3:** Bilateral combinations (COMB) of the types of origin of the ascending pharyngeal artery. R: right; L: left. *N* = 85 patients.

COMB R/L	Count	%
**0-0**	11	12.94
**I-0**	1	1.18
**I-II**	19	22.35
**I-I**	8	9.41
**I-III**	4	4.71
**II-0**	1	1.18
**II-I**	4	4.71
**II-II**	8	9.41
**II-V**	1	1.18
**II-III**	7	8.24
**IV-I**	1	1.18
**IV-II**	5	5.88
**IV-III**	2	2.35
**V-II**	1	1.18
**III-0**	1	1.18
**III-II**	7	8.24
**III-III**	4	4.71

**Table 4:** Different S-types of external carotid branch sequence associate with an occipitopharyngeal trunk (OPT). Count and prevalence (%) in the general lot (*N* = 170) and on the sides. R: right side; L: left side.

‘S’-Types with OPT	General Lot (*N* = 170)		R (*N* = 85)		L (*N* = 85)	
	Count	%	Count	%	Count	%
10	9	5.29	5	5.88	4	4.71
17	3	1.76	-	-	3	3.53
20	1	0.59	-	-	1	1.18
25	3	1.76	1	1.18	2	2.35
37	1	0.59	-	-	1	1.18
6	1	0.59	6	7.06	1	1.18
9	11	6.47	-	-	5	5.88

**Table 5:** Prevalence (%) and count of cases with different ‘S’ types of external carotid artery’s branches origin sequence in the overall group (*N* = 170), right side (R, *N* = 85), and left side (L, *N* = 85). S0: ascending pharyngeal artery absent or with internal carotid origin.

‘S’ Type	Bilateral (*N* = 170)		Right Side (*N* = 85)		Left Side (*N* = 85)	
	*%*	*Count*	*%*	*Count*	*%*	*Count*
**1**	1.76	2	1.18	1	1.18	1
**2**	7.06	7	5.88	5	2.35	2
**3**	7.06	6	2.35	2	4.71	4
**4**	0.59	9	5.88	5	4.71	4
**5**	1.18	1			1.18	1
**6**	1.18	1	1.18	1		
**7**	4.12	12	5.88	5	8.24	7
**8**	5.29	11	5.88	5	7.06	6
**9**	3.53	12	7.06	6	7.06	6
**10**	0.59	10	4.71	4	7.06	6
**11**	0.59	1	1.18	1		
**12**	2.35	4	2.35	2	2.35	2
**13**	1.18	2			2.35	2
**14**	5.88	5	4.71	4	1.18	1
**15**	2.94	3	3.53	3		
**16**	1.18	2			2.35	2
**17**	1.76	3	3.53	3		
**18**	1.18	2			2.35	2
**19**	4.12	2	1.18	1	1.18	1
**20**	2.94	2	2.35	2		
**21**	6.47	7	4.71	4	3.53	3
**22**	0.59	5	2.35	2	3.53	3
**23**	1.18	2			2.35	2
**24**	0.59	1	1.18	1		
**25**	1.18	3	2.35	2	1.18	1
**26**	1.18	4			4.71	4
**27**	0.59	2	1.18	1	1.18	1
**28**	1.76	2	1.18	1	1.18	1
**29**	2.35	2	2.35	2		
**30**	0.59	1			1.18	1
**31**	0.59	1	1.18	1		
**32**	0.59	1	1.18	1		
**33**	0.59	1	1.18	1		
**34**	0.59	1			1.18	1
**35**	0.59	1			1.18	1
**36**	1.18	1	1.18	1		
**37**	0.59	1	1.18	1		
**38**	1.18	2	1.18	1	1.18	1
**39**	0.59	1	1.18	1		
**40**	19.41	1	1.18	1		
**S0**	**1.18**	**33**	**16.47**	**14**	**22.35**	**19**

**Table 6:** Prevalence (%) and count of cases with different ‘S’ types of external carotid artery’s branches origin sequence by gender and side. S0: ascending pharyngeal artery absent or with internal carotid origin.

‘S’ Type	Males (*N* = 54), Right Side		Males (*N* = 54), Left Side		Females (*N* = 31), Right Side		Females (*N* = 31), Left Side	
	*%*	*Count*	*%*	*Count*	*%*	*Count*	*%*	*Count*
**1**	1.85	1					3.23	1
**2**	3.70	2	5.56	3			6.45	2
**3**	5.56	3	1.85	1	3.23	1	3.23	1
**4**	5.56	3	9.26	5	3.23	1		
**5**					3.23	1		
**6**							3.23	1
**7**	9.26	5	5.56	3	6.45	2	6.45	2
**8**	5.56	3	3.70	2	9.68	3	9.68	3
**9**	9.26	5	9.26	5	3.23	1	3.23	1
**10**	5.56	3	5.56	3	9.68	3	3.23	1
**11**			1.85	1				
**12**			1.85	1	6.45	2	3.23	1
**13**	1.85	1			3.23	1		
**14**	1.85	1	5.56	3			3.23	1
**15**			5.56	3				
**16**	1.85	1			3.23	1		
**17**			5.56	3				
**18**	1.85	1			3.23	1		
**19**					3.23	1	3.23	1
**20**							6.45	2
**21**	5.56	3	7.41	4				
**22**	5.56	3	1.85	1			3.23	1
**23**	1.85	1			3.23	1		
**24**							3.23	1
**25**					3.23	1	6.45	2
**26**	1.85	1			9.68	3		
**27**	1.85	1					3.23	1
**28**			1.85	1	3.23	1		
**29**			1.85	1			3.23	1
**30**	1.85	1						
**31**			1.85	1				
**32**			1.85	1				
**33**			1.85	1				
**34**	1.85	1						
**35**	1.85	1						
**36**			1.85	1				
**37**							3.23	1
**38**					3.23	1	3.23	1
**39**			1.85	1				
**40**			1.85	1				
**S0**	24.07	13	14.81	8	19.35	6	19.35	6

**Table 7:** Origins of the ascending pharyngeal artery (APA). ECA: external carotid artery; CCA: common carotid artery; OA: occipital artery; ICA: internal carotid artery; FA: facial artery; LA: lingual artery; LFT: linguofacial trunk; CB: carotid bifurcation; OPT: occipitopharyngeal trunk. * Based on 2 cases out of 308 examined specimens across all published studies [28].

Origin	Frequency	Anatomical Characteristics	References
ECA	66–81% (most common)	Arises from the posteromedial aspect, usually the first or second branch near the carotid bifurcation	[5,8,23,33,35]
CCA/CB	1–10%	CCA near the CB, or CB	[5,32,33,35,11]
OA	~2%	OPT from ECA	[5,8,23]
ICA	1.4–8%	Isolated cases, clinically significant for surgical planning, potential hazard during carotid endarterectomy	[5,8,12,25,31,33,37,38,39]
FA	~2% (rare)	Variant origin	[5,8]
LA	0.5%	Variant origin, LA-APA trunk	[33,40]
LFT (LA-FA-APA trifurcation)	extremely rare (0.65%) *	APA arises from a common trunk with lingual and facial arteries; only 1 cadaveric case has been previously reported, the first CTA-documented case in 2025	[5,28]
OA (arising from ICA)	extremely rare (case report)	APA originated from the OA; the OA arose from a common trunk with STA from the cervical segment of the ICA. Associated with low CB and ICA occlusion.	[41]
superior laryngeal artery	0.5%	Variant origin	[33]
ascending cervical artery	extremely rare	In such cases, a false absence of the APA, or an APA occluded by an extensive carotid plaque, may be suspected.	[1]

**Table 8:** Comparison of the prevalence of the ascending pharyngeal artery origin by various authors. Abbreviations: ECA = external carotid artery; ECA-M = ECA medial wall; ECA-P = ECA posterior wall; OA = occipital artery; OPT = occipitopharyngeal trunk; ICA = internal carotid artery; CCA = common carotid artery; CB = carotid bifurcation; FA = facial artery; LA = lingual artery; LFT = linguofacial trunk; 3D-RA = three-dimensional rotational angiography; angio-CT = angiographic computed tomography. * Akiyama et al. reported duplication of APA in 1.4% of cases (4/277). Note: Cobiella et al. CCA/CB included both CCA (1%) and CB (9.7%) origins. (-): indicates data not reported or not specified in the study; “rare” indicates cases reported without a specific percentage.

Authors	*N* (Sides)	ECA Total	ECA-M	ECA-P	OA/OPT	ICA	CCA/CB	FA	LA/LFT	Absent
Present Study	170	62.35%	26.47%	35.88%	17.06%	4.71%	1.18%	-	1.18%	14.71%
Akiyama et al., 2025 [23]	277	60.1%	-	-	34.8%	3.3%	1.5%	-	0.4%	1.4% *
Cobiella et al., 2021 [33]	207	70.5%	-	-	13.5%	1.4%	10.7%	rare	rare	-
Bergman et al., 1988 [42]	-	65–80%	-	-	14–20%	-	7–9%	-	-	-
Lippert and Pabst, 1985 [8]	-	70%	-	-	20%	8%	-	2%	-	-
Lasjaunias and Moret, 1976 [44]	-	65%	-	-	-	-	-	-	-	-
Cavalcanti et al., 2009 [5]	20	80%	56%	44%	-	-	-	-	-	20%

**Table 9:** Comparison of double/duplicate ascending pharyngeal artery findings. Abbreviations: APA = ascending pharyngeal artery; ECA = external carotid artery; ICA = internal carotid artery; CEA = carotid endarterectomy; LFT = linguofacial trunk; CNI = cranial nerve injury; 3D-RA = three-dimensional rotational angiography; Angio-CT = angiographic computed tomography; SCM = sternocleidomastoid; OR = odds ratio.

Parameter	Akiyama et al., 2025 [23]	Calotă et al., 2025 [29]
**study** **design**	prospective case series with 3D rotational angiography	case report with angio-CT imaging
**sample size**	279 carotid arteries (259 patients undergoing CEA)	2 adult males (aged 65 years each)
**imaging method**	3D-RA (three-dimensional rotational angiography)	angio-CT (32-slice CT scanner, 0.6 mm collimation)
**prevalence of duplication**	4/279 cases (1.4%)	2/2 cases (100%—case report)
**classification**	not specified—reported as “duplication”	distinguished: (1) true duplication (Case 1), (2) two-rooted/partly duplicated (Case 2)
**types of duplication**	both APAs from ECA: 2 cases; one from ECA + one from occipital artery: 1 case; one from ECA + one from ICA: 1 case.	case 1: Both APAs from the ECA (inferior infrahyoid + superior suprahyoid); case 2: Two roots from the ECA posterior wall converging to a single trunk.
**side affected**	not specified per case	case 1: Right side (double); case 2: Left side (two-rooted).
**relationship to the hyoid**	not reported	case 1: inferior APA infrahyoid, superior APA 0.69 cm suprahyoid; case 2: both roots suprahyoid.
**associated anatomical features**	not specified	case 1: LFT with infrahyoid origin; case 2: anastomotic branch to ascending palatine artery; superiorly looped SCM branch.
**clinical context**	patients undergoing CEA; emphasis on surgical manipulation and CNI risk	incidental findings on angio-CT; emphasis on surgical landmarks and bleeding risk
**APA manipulation required**	required in 15.8% of all CEA cases; duplication cases not analyzed separately	not applicable (case report, no surgical intervention)
**key clinical implications**	higher CNI risk when APA is manipulated (OR 5.70, p = 0.086); more frequent in high carotid bifurcation; preoperative 3D-RA recommended.	risk of persistent bleeding if one trunk is unrecognized; the greater hyoid horn is not a reliable landmark; both trunks must be identified in surgery; preoperative imaging is critical.
**novel findings**	the first systematic documentation of APA duplication prevalence in the CEA population	the first detailed anatomical description distinguishing true duplication from a two-rooted variant

**Table 10:** Embryological correlations of the anatomic variations of the ascending pharyngeal artery (APA). OPT: occipitopharyngeal trunk; LFT: linguofacial trunk; TLT: thyrolingual trunk.

Variation Type	Embryological Mechanism
**Type** **0 (Absent APA)**	Complete regression of the dorsal aortic root cranial extension
**Type IV (ICA origin)**	Persistence of APA–third arch connection (normally disappears)
**Common trunks (OPT, LFT, TLT)**	Incomplete separation of adjacent pharyngeal sprouts; shared embryonic origin
**Variable sequential order**	Differential timing of pharyngeal artery sprouting and regression
**Double APA**	Separate insertion of the middle/inferior pharyngeal branches with incomplete fusion
**Infrahyoid origins**	Persistence of lower ventral pharyngeal connections

**Table 11:** Terminal branching variations of the external carotid artery (ECA). MA: maxillary artery; MMA: middle meningeal artery; PAA: posterior auricular artery; STmA: superficial temporal artery; TFA: transverse facial artery.

Pattern	Terminal Branches	Frequency	References
normal bifurcation (type I)	MA + STmA	~90–95% (most common)	[71,74]
terminal trifurcation (type IIA)	MA + STmA + TFA	rare (~10% of variations)	[71]
terminal trifurcation (type IIB)	MA + STmA + MMA	extremely rare (single case reports)	[71,72]
terminal trifurcation (type III)	MA + STA + PAA	very rare	[72]
terminal pentafurcation	APA + FA + OA + MA + STmA	extremely rare (first reported 2021)	[72]
maxillofacial trunk (type IV)	ECA continues as STmA; MA + FA arise as a common trunk	rare	[75,76]
ECA trifurcation (type IIB) with PAA from STmA	variant MA + STmA + MMA	case report	[72]

## Data Availability

The original contributions presented in this study are included in the article. Further inquiries can be directed to the corresponding author.

## Abbreviations

The following abbreviations are used in this manuscript:

abs	absent
APA	ascending pharyngeal artery
CB	carotid bifurcation
CCA	common carotid artery
CEA	carotid endarterectomy
ECA	external carotid artery
FA	facial artery
ICA	internal carotid artery
IJV	internal jugular vein
IPS	inferior petrosal sinus
LA	lingual artery
LFT	linguofacial trunk
OA	occipital artery
OPT	occipitopharyngeal trunk
SCM	sternocleidomastoid
STA	superior thyroid artery
TLT	thyrolingual trunk
